# Cytotoxic Potential of the Marine Diatom *Thalassiosira rotula*: Insights into Bioactivity of 24-Methylene Cholesterol

**DOI:** 10.3390/md20100595

**Published:** 2022-09-23

**Authors:** Adele Cutignano, Mariarosaria Conte, Virginia Tirino, Vitale Del Vecchio, Roberto De Angelis, Angela Nebbioso, Lucia Altucci, Giovanna Romano

**Affiliations:** 1 Institute of Biomolecular Chemistry, National Research Council, Via Campi Flegrei 34, 80078 Pozzuoli, Italy; 2 Department of Ecosustainable Marine Biotechnology, Stazione Zoologica Anton Dohrn, Via Akton 55, 80133 Napoli, Italy; 3 Department of Precision Medicine, University of Campania ‘L. Vanvitelli’, Via L. De Crecchio 7, 80138 Napoli, Italy; 4 Department of Experimental Medicine, Section of Biotechnology, Molecular Medicine and Medical Histology, University of Campania “L. Vanvitelli”, Via L. de Crecchio 7, 80138 Napoli, Italy; 5 Biogem, Institute of Molecular Biology and Genetics, Via Camporeale Area P.I.P., 83031 Ariano Irpino, Italy

**Keywords:** diatoms, *Thalassiosira rotula*, phytosterols, 24-methylene cholesterol, cytotoxicity, cancer, proapoptotic compounds, cell cycle arrest

## Abstract

Marine microalgae are receiving great interest as sustainable sources of bioactive metabolites for health, nutrition and personal care. In the present study, a bioassay-guided screening allowed identifying an enriched fraction from SPE separation of the methanolic extract of the marine diatom *Thalassiosira rotula* with a chemically heterogeneous composition of cytotoxic molecules, including PUFAs, the terpene phytol, the carotenoid fucoxanthin and the phytosterol 24-methylene cholesterol (24-MChol). In particular, this latter was the object of deep investigation aimed to gain insight into the mechanisms of action activated in two tumour cell models recognised as resistant to chemical treatments, the breast MCF7 and the lung A549 cell lines. The results of our studies revealed that 24-MChol, in line with the most studied β-sitosterol (β-SIT), showed cytotoxic activity in a 3–30 µM range of concentration involving the induction of apoptosis and cell cycle arrest, although differences emerged between the two sterols and the two cancer systems when specific targets were investigated (caspase-3, caspase-9, FAS and TRAIL).

## 1. Introduction

**Figure 1:**
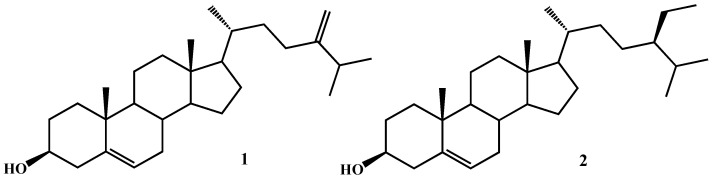
Chemical structures of 24-methylene cholesterol (**1**) and β-sitosterol (**2**).

Microalgae are a large and diverse group of photosynthetic microorganisms that populate marine and freshwater environments. In the last years, they have been attracting the attention of scientific communities due to the potentially sustainable production of their bioactive metabolites, including carotenoids, polysaccharides, glycolipids, polyunsaturated fatty acids (PUFAs) and their metabolites, which can find applications in the food, cosmetic and pharmaceutical industries. In fact, originally isolated in their environment, single microalgal species can be cultivated on a large scale, thus allowing overcoming the supply issue that often constitutes one of the main problems in the exploitation of natural products for human well-being and health. High-throughput screening programs conducted during our research activity in the past years on microalgal extracts gave a number of positive hits evidencing an array of biological properties, ranging from antibacterial to antifungal, immunomodulant and cytotoxic activities [1,2,3,4,5,6,7]. As for the latter, we recently reported the structural identification of a class of glycerolipids, namely monoacylglycerols (MAG) of C16/C20 unsaturated fatty acids in the marine diatom *Skeletonema marinoi* and their cytotoxic and proapoptotic activity on specific tumour cells, i.e., HCT-116 and U-937 through the activation of caspase-3/7 [4]. A literature survey revealed that the cytotoxicity of microalgal extracts is associated with diverse chemicals, including carotenoids such as fucoxanthin [8,9,10], sulphated polysaccharides [11], glycoglycerolipids such as monogalactosyldiacylglycerols (MGDG) [12], PUFAs (especially EPA and DHA) [13] and oxylipins [14,15], acting by different mechanisms not always fully elucidated, thus suggesting that a single microorganism may be a treasure chest of pharmacologically promising molecules. In the framework of the Italy–South Africa joint research project: ‘Genomics for a blue economy’, during a preliminary bioactivity screening on the human breast cancer cell line MCF7 [16], we selected a marine diatom species, *Thalassiosira rotula*, and focused our studies on the cytotoxic properties of its methanolic extracts, aiming at identifying the molecules responsible for the observed bioactivity. Following a bioassay-guided fractionation approach, we achieved the identification of a single fraction containing different metabolites with cytotoxic activity against a selected tumour cell line. Here, we report an in-depth investigation on antiproliferative selective activity and the underlying mechanism of action of one of the bioactive microalgal compounds, the 24-methylene cholesterol (24-MChol, **1**, Figure 1), on a panel of cancer cells in comparison with β-sitosterol (β-SIT, **2**, Figure 1), one of the most common and studied plant phytosterols.

## 2. Results and Discussion

### 2.1. Bioassay-Guided Fractionation of Microalgal Extract

**Figure 2:**
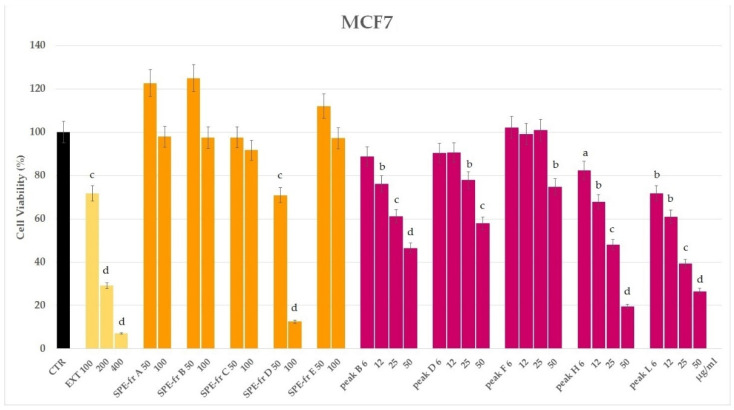
Cell viability by MTT assay of the *Thalassiosira rotula* methanolic extract (EXT); SPE fractions (A–E) and HPLC peaks (B, D, F, H, and L) on the MCF7 cell line after 24-h exposure. Statistical notations: a = *p* ≤ 0.05, b = *p* ≤ 0.01, c = *p* ≤ 0.001 and d = *p* ≤ 0.0001.

The methanolic extract of the cell pellet of the marine diatom *Thalassiosira rotula* was preliminary tested by the MTT assay on the MCF7 cell line at a concentration of 100, 200 and 400 µg/mL. Remarkable cytotoxic activity was evidenced at 200 µg/mL after 24 h and at 100 µg/mL after 48 h (Figure 2 and Supplementary Figures S1 and S2).

Prompted by these results, and in order to get an indication on the chemical nature of the bioactive compound(s), we proceeded with the further purification of an aliquot of the extract (43.7 mg) by HRX-SPE cartridge using a method we previously developed for the fractionation of marine extracts [1]. According to this protocol, five fractions were obtained eluting with solvents by increasing the lipophilicity. Fraction D (3.2 mg), eluted by acetonitrile, exhibited cytotoxicity towards MCF7 cells, reducing the cell viability to 13% at 100 µg/mL with an enhanced activity compared to the original extract (72% at 100 µg/mL) (Figure 2).

**Figure 3:**
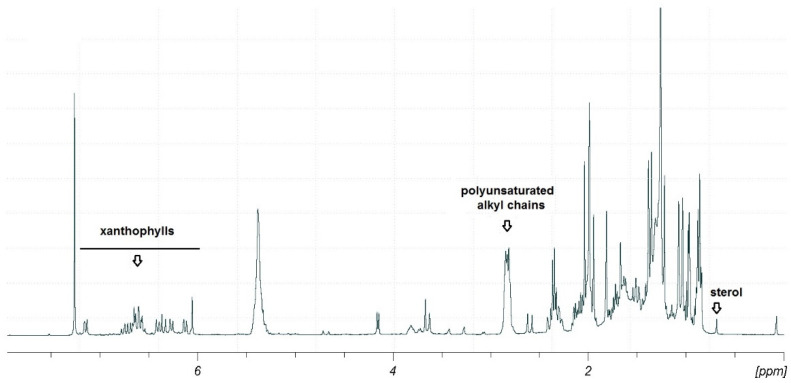
^1^H-NMR spectrum (400 MHz, CDCl_3_) of fraction D after SPE fractionation of the methanolic extract of *Thalassiosira rotula*. The key signals for xanthophylls, polyunsaturated alkyl chains and sterols have been marked.

Combined information gathered by the preliminary TLC, LC-MS/MS and ^1^H NMR analyses suggested the occurrence in this fraction mainly of polyunsaturated fatty acids (PUFAs), xanthophylls and sterols (Figure 3).

### 2.2. Chemical Identification of Bioactive Metabolites

In order to isolate a sufficient amount of pure bioactive metabolite(s) for chemical and biological characterisation, pooled D fractions (9.5 mg) from three parallel HRX-SPE fractionations were purified by RP-HPLC. The collected subfractions were tested on MCF7 cells, and cytotoxicity was shown in five (B, D, F, H and L) out of eleven peaks (Figure 2 and Supplementary Figures S1 and S2). Peak B (Retention time (Rt) = 2.7 min), analysed by direct ^1^H NMR, resulted in a mixture of fatty acids, including polyunsaturated derivatives (PUFAs). Peak D (Rt = 7.5 min) exhibited an UV profile distinctive of xanthophylls with a maximum wavelength absorbance at 450 and 468 nm. In fact, after solvent removal, it gave an orange amorphous solid that was plainly identified by ^1^H NMR and HR-ESIMS/MS (M + Na^+^ *m/z* 681.4132) as fucoxanthin, a typical microalgal carotenoid (Supplementary Figures S3 and S4). Peak F (Rt = 13.6 min) was identified by 1D and 2D-NMR and GC-MS (M^+^ *m/z* 296.06) as the linear diterpene alcohol phytol (Supplementary Figures S5 and S6). The ^1^H NMR spectrum of peak H (Rt = 16.4 min) showed signals attributable to a polyunsaturated hydrocarbon skeleton (Supplementary Figure S7). An old report attests to the occurrence of all-(*Z*)-heneicosa-3,6,9,12,15,18-hexaene (HEH) in several microalgal species, including *Thalassiosira* sp. [17]. However, this product quickly degraded in the NMR tube, preventing further chemical and biological investigations; thus, its identification as HEH, related hydrocarbons [18,19] or other polyunsaturated compounds remains yet undetermined. Finally, peak L eluting at Rt = 25.1 min was identified as 24-methylene cholesterol (24-MChol, **1**, Figure 1) by ^1^H-NMR and GC-MS (M^+^ *m/z* 398.26) in comparison with a reference standard (Supplementary Figures S8 and S9). This sterol is the most abundant in the microalgal extract and has been reported in diatoms of the genus *Thalassiosira* and in the species *T. rotula* [20,21]. Considering the large wealth of studies on the cytotoxic activity of PUFAs, fucoxanthin and phytol on cancer cells [9,10,13,22], we decided to focus our attention on the phytosterol derivative **1,** which showed cytotoxic activity towards MCF7 cells with an IC_50_ = 17.5 μg/mL (Supplementary Figure S1).

Phytosterols (PS) are a group of sterols structurally similar to animal cholesterol but exclusively found in higher plants, algae and microalgae. The structural differences occur at the lateral chain and include additional methyl or ethyl groups and/or double bonds. PS have been mainly studied for their properties of lowering cholesterol levels in humans by reducing its absorption from the gut, thus offering protection against cardiovascular disease. In more recent years, they have also been reported to show cytotoxic activity on several tumour cell lines, thus stimulating the interest toward their possible use in cancer therapies or prevention [23,24,25,26,27,28]. Yet, little is known about the mechanisms underlying the cytotoxic effects. PS may exert cytotoxicity through multiple mechanism of actions, i.e., affecting cell cycle progression [25,26,28,29] and inducing cellular apoptosis [25,26,28]. In addition, PS may reduce angiogenesis and tumour metastasis by inhibiting cell migration [30]. The majority of studies have been carried out on β-sitosterol (β-SIT, **2**, Figure 1), the most common plant sterol [29,31,32,33,34]; interestingly, experimental observation suggested that this sterol may stimulate the sphingomyelin cycle via receptors belonging to the tumour necrosis factor superfamily (i.e., FAS), activating sphingomyelinase or the de novo biosynthesis of ceramide and increasing the levels of this endogenous proapoptotic molecule acting via caspase-3/9 [23,35,36].

In diatoms, more than forty different sterols have been identified, eleven of which were in the most abundant across more than one hundred species [37]. Although a sterol composition cannot be used as a biomarker, Thalassiosiraceae typically contain high relative abundances of 24-MChol, which indeed is one of the major diatom sterols. Yet, to the best of our knowledge, 24-MChol was not specifically investigated for its anticancer properties.

### 2.3. Bioactivity Studies

To explore the effect of 24-MChol on cell proliferation, we performed a preliminary screening on a panel of cancer cells, including the breast MCF7, the colon SW480, the lung A549 and the human histiocytic lymphoma U937 cancer cell lines compared to MePR2B normal cells. The cell proliferation rates were monitored up to 48 h after treatment at the 5–50-μM concentration range. Based on the dynamic monitoring of cell proliferation by XCELLigence, we decided to select for further assays the A549 (Supplementary Figure S10) and MCF7 (Supplementary Figure S11) cell lines, both generally resistant to chemical treatment [16,38], which here showed a higher susceptibility to 24-MChol in a time- and concentration-dependent manner; furthermore, the A549 cell line has never been tested for a response at the PS treatment; conversely, SW480 cells were resistant to PS treatments (Supplementary Figure S12) [39,40]. U-937 cell proliferation following the treatment with PS was also monitored (Supplementary Figure S13). This cell line was also very sensitive at lower dosages, and indeed, it is targeted by many different treatments, as many metabolic-related genes are involved in leukaemia [41]. MePR2B normal cells were used as the control cell line to prove anticancer specificity. Since the MePR2B primary cell line was immortalised from human amniocytes, the proliferation was monitored at earlier times [42] (Supplementary Figure S14).

**Figure 4:**
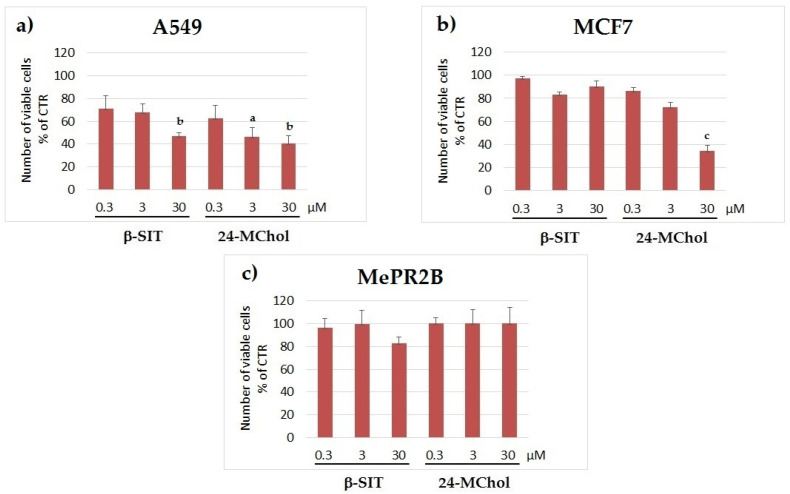
Cell proliferation rates in the (**a**) A549, (**b**) MCF7 and (**c**) MePR2B cell lines after treatment with β-SIT or 24-MChol at concentrations of 0.3, 3 and 30 μM, observed after 24 h. Statistical notations: a = *p* ≤ 0.05, b = *p* ≤ 0.01 and c = *p* ≤ 0.001.

For successive assays on the selected cell lines, 24-MChol was used at increasing concentrations (0.3, 3 and 30 μM) and compared to β-SIT as the reference compound, which was previously tested on MCF7 cells [31,34,41]. The proliferation rates by the trypan blue exclusion method showed a strong decrease of cell proliferation in both the A549 and MCF7 cell lines in a dose-dependent manner following 24–48 h of treatment with no impact on MePR2B cells after 24 h, suggesting the presence of a therapeutic window for the cancer-specific activity of PS (Figure 4 and Supplementary Figure S15).

Afterwards, we evaluated the effect of 24-MChol versus β-SIT on cell viability by using the MTT assay. The results showed that 24-MChol had an inhibitory effect on the viability of both tumour cell lines tested in a dose-dependent manner after 24 h. In particular, MCF7 cells were more sensitive to 24-MChol treatment than A549 at the lowest concentration. This difference became relevant at 3 μM (19% of cell death increasing in MCF7 vs. A549), but it regressed at 30 μM, where the effectiveness of the treatments was very similar, with a viability decrease of about 70% (Supplementary Figure S16). Conversely, in the MePR2B cell line, 24-MChol did not show any cytotoxic effect. The treatment with β-SIT showed a significant effect on MCF7 cell viability at 30 μM, but it was less effective compared to 24-MChol.

**Figure 5:**
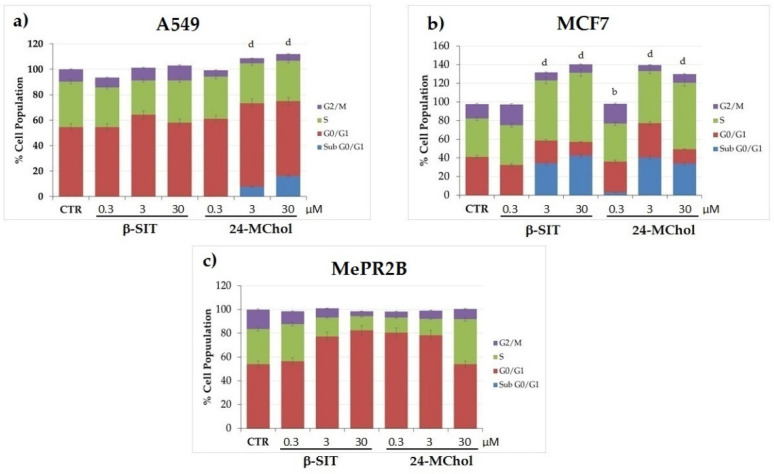
Cell cycle analysis on the (**a**) A549, (**b**) MCF7 and (**c**) MePR2B cell lines after 24 h of treatment with β-SIT and 24-MChol at 0.3–3 and 30 μM. Statistical notations for differences in the cell populations in subG0/G1 compared to the CTR: b = *p* ≤ 0.01 and d = *p* ≤ 0.0001.

In order to gain deeper insights into the effects on the cell cycle, MCF7 and A549 cells were treated with 24-MChol or β-SIT for 24 h at concentrations of 0.3, 3 and 30 μM (Figure 5 and Supplementary Figure S17). A cell cycle analysis of A549 showed that the subG0/G1 peaks did not change following the β-SIT treatments. On the other hand, after the 24-MChol treatments, we observed 7% and 16% of the subG0/G1 cell populations at the 3 and 30-μM concentrations, respectively. In MCF7 cells, we noticed that 24-MChol induced cell death with a S-phase cell cycle arrest at the 3 and 30-μM concentrations (respectively, 16% and 31% of the S-phase cell population increased) and the presence of a subG0/G1 peak (respectively, 40% and 33% of the subG0/G1 cell population). On the contrary, it could not detect any difference in the MePR2B cell cycle at all the concentrations tested. The β-SIT treatment was significantly effective only in MCF7 cells, with the presence of a subG0/G1 peak at 3 and 30 μM (respectively, 34% and 42% of the cell population), as already reported [31]. These results suggest the ability of 24-MChol to induce cell death in both cancer cell lines differently from β-SIT that does not affect A549 cells (Figure 5). These results corroborate our previous findings reported in Supplementary Figures S10, S11 and S14, in which 24-MChol induces a cell proliferation arrest in both cancer cell lines without affecting the MePR2B normal cell line.

**Figure 6:**
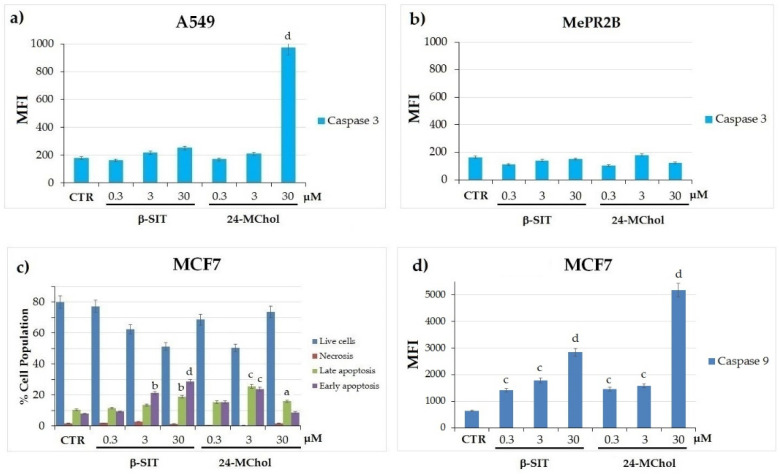
(**a**) Caspase-3 activity assay by CaspGLOW Fluorescein Active Caspase-3 in the A549 and (**b**) MePR2B cell lines treated with β-SIT or 24-MChol in the range of 0.3–30 µM. Caspase-3 activity was measured using FITC fluorochrome and calculated as the mean fluorescence intensity (MFI) for each sample. (**c**) Annexin V/PI staining by the FITC Annexin V Apoptosis Detection Kit in MCF7 cells treated with β-SIT or 24-MChol in the range of 0.3–30 µM. (**d**) Caspase-9 assay by the SR-FLICA^®^ Caspase-9 Assay Kit in MCF7 cells treated with β-SIT or 24-MChol in the range of 0.3–30 µM. Caspase-9 activity was measured using PE fluorochrome and calculated as the mean fluorescence intensity (MFI) for each sample. Statistical notations for differences in the apoptotic cell populations compared to the CTR apoptotic cells: a = *p* ≤ 0.05, b = *p* ≤ 0.01, c = *p* ≤ 0.001 and d = *p* ≤ 0.0001.

To better evaluate the cell death process induced by 24-MChol, we performed a caspase-3 activity assay in the A549 and MePR2B cell lines and annexin V/PI staining and caspase-9 assays in MCF7 cells (Figure 6). In A549 cells, we demonstrated that 24-MChol induced high levels of caspase-3 activity at a 30-μM concentration (MFI = 970 vs. control MFI = 179), confirming the results obtained with the cell cycle analysis. On the contrary, the β-SIT treatment did not affect A549 and MePR2B cells (Figure 6a,b). Since MCF7 cells do not express caspase-3 [43], we performed Annexin/PI and caspase-9 assays on this cell line. This allowed us to reveal cells in late and early apoptosis, in necrosis and viable cells by detecting phosphatidylserine exposed at the cell surface and loss of the membrane permeability. The cells marked with double staining were in late apoptosis. The cells marked only with annexin V were in early apoptosis. Necrotic cells show only PI red fluorescence, while live cells show no annexin V or PI fluorescence. In our setting, after the 24-MChol treatment, we observed a strong apoptotic effect—in particular, at a 3-μM concentration—with about 30% of the apoptotic cell population increasing. As previously reported [31,33], MCF7 cells are sensitive to β-SIT, and we detected a significant apoptotic effect in these cells at 3 and 30 μM (respectively, 16% and 29% of the apoptotic cell population increased) (Figure 6c and Supplementary Figure S18). We also observed that both β-SIT and 24-MChol induced an increase in caspase-9, with a stronger effect induced by 24-MChol at 30 μM (MFI 5177 vs. control MFI 646) (Figure 6d). These data confirmed that 24-MChol induced apoptosis in both cell lines, while β-SIT was only effective in MCF7.

Anticancer activity of PS may affect different molecular pathways, which, in turn, are dysregulated, depending on the specific tumour microenvironment. Starting from this premise, and to study the specific biological mechanism activated by 24-MChol, we performed a Western blot analysis on the breast and lung cancer systems to better understand the impact on apoptosis, a crucial cellular pathway involved in the pathophysiology of cancer development and progression. Since FAS and TRAIL receptors are well-known drivers of tumour proliferation, we investigated whether the treatment with β-SIT and/or 24-MChol might have a responsive effect in our cell models.

**Figure 7:**
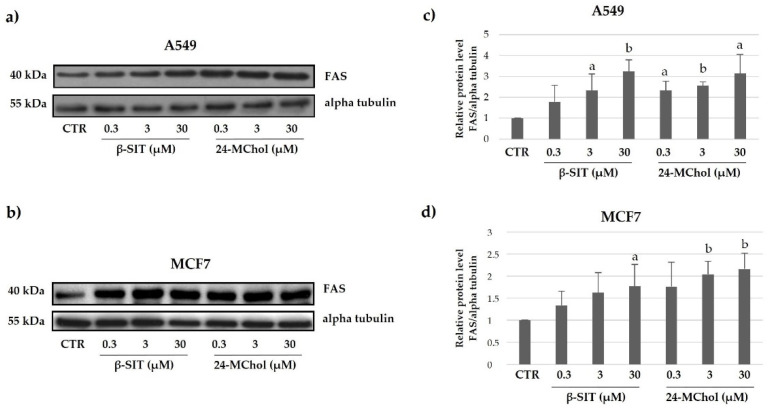
Representative Western blot analysis for FAS in (**a**) A549 and (**b**) MCF7 cells after treatment with β-SIT and 24-MChol at increasing concentrations (0.3–30 μM). Alpha tubulin was used as the loading control. The intensity of the bands was measured by ImageJ software and reported in the graphs (**c**) and (**d**) as the means and SD of three biological replicates. Statistical notations: a = *p* ≤ 0.05 and b = *p* ≤ 0.01.

FAS is a member of the tumour necrosis factor receptor (TNF-R) superfamily, which also includes receptors for TNFα, TRAIL (TNF-related apoptosis-inducing ligand), receptor activator of NF-kB ligand (RANKL), CD40 ligand (CD40L) and other members of the TNF family of cytokines. Specifically, FAS belongs to the subgroup of TNF- R family members that have an intracellular death domain. FAS can initiate apoptosis and is important in both the pathological and physiologic conditions. In our models, after 24 h of treatment, we observed a strong increase in the FAS protein in both MCF7 and A549 cell lines (Figure 7 and Supplementary Figures S19 and S20). These results confirm and reinforce our hypothesis that 24-MChol is able to activate the apoptosis pathway.

**Figure 8:**
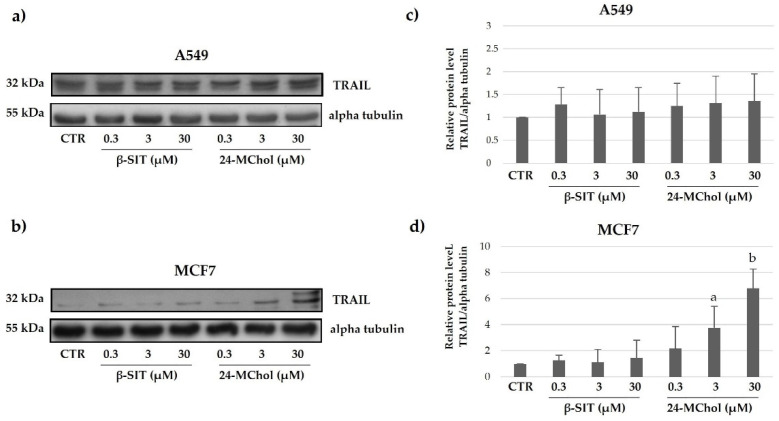
Representative Western blot analysis for TRAIL in (**a**) A549 and (**b**) MCF7 cell lines after treatments with β-SIT and 24-MChol at increasing concentrations (0.3–30 μM). Alpha tubulin was used as the loading control. The intensity of the bands was measured by ImageJ software and reported in the graphs (**c**) and (**d**) as the means and SD of three biological replicates. Statistical notations: a = *p* ≤ 0.05 and b = *p* ≤ 0.01.

Another important stimulator of apoptosis is TRAIL. Tumour cells are significantly more sensitive to TRAIL-induced apoptosis than normal cells. Although the molecular basis for the tumour-selective activity of TRAIL remains to be fully defined, the TRAIL pathway is an attractive therapeutic target for the treatment of cancer. TRAIL interactions with its receptors can result in the activation of either extrinsic or intrinsic apoptosis pathways in tumour cells. We found that the expression levels of TRAIL in the A549 and MCF7 cell lines after treatment with β-SIT and 24-MChol at increasing concentrations were different (Figure 8 and Supplementary Figures S21 and S22). In particular, the data showed an increase in the TRAIL levels for MCF7 cells after the β-SIT and 24-MChol treatments. On the other hand, no change is detectable for A549 cells. This could be due to a different mechanism in apoptosis activation. Thus, it seems that following the treatment with 24-MChol, in MCF7, the activation of the apoptotic pathway is TRAIL-dependent, whereas A549 cells undergo to an activation of apoptosis independent from TRAIL activity [44].

## 3. Materials and Methods

### 3.1. General

One-dimensional and two-dimensional NMR spectra were recorded on a Bruker AVANCE™ III HD-400 spectrometer equipped with a CryoProbe™ Prodigy and on a Bruker DRX-600 equipped with a TXI CryoProbe^TM^ in CDCl_3_ or CD_3_OD (δ_H_ values reported refer to the residual solvent protons at 7.26 and 3.34 ppm, respectively; δ_C_ values refer to solvent carbons at 77.0 and 49.0 ppm, respectively). High-resolution mass spectra were acquired on a Q-Exactive Hybrid Quadrupole-Orbitrap Mass Spectrometer (Thermo Scientific, Milan, Italy) online with the UHPLC apparatus Infinity 1290 (Agilent Technologies, Santa Clara, CA, USA). HPLC analyses were performed on a Shimadzu high-performance liquid chromatography system (Shimadzu, Kyoto, Japan) LC-20ADXR equipped with a Diode Array Detector SPDM-20A and a LUNA C-18(2) column 250 × 4.6 mm, 5 µm, 100A (Phenomenex, Castel Maggiore, Italy). GC-MS analyses were performed on an ion-trap MS instrument in EI mode (70eV) (Thermo, Polaris Q) connected with a GC system (Thermo, GCQ) by a 5% phenyl/methyl polysiloxane column (30 m × 0.25 mm × 0.25 µm, Agilent, VF-5ms) using helium as the gas carrier. TLC plates (KieselGel 60 F254) and silica gel powder (KieselGel 60, 0.063–0.200 mm) were from Merck (Darmstadt, Germany). Chemicals were of analytical reagent grade and were used without any further purification. Standard compounds 24-methylene cholesterol and β-sitosterol were purchased from Merck (Darmstadt, Germany). All solvents were of HPLC and LCMS grade (VWR International, Milan, Italy).

### 3.2. Microalgal Culturing and Biomass Harvesting

*T. rotula*, strain CCMP1647 (SZN strain code: FE80), was isolated in 2011 in the Gulf of Naples (40°48.5′ N, 14°15′ E), the Mediterranean Sea. Clonal cultures were established by isolating single cells from phytoplankton net samples collected from the surface layer of the water column. Cultures were grown in 10 l Nalgene transparent carboys (Fisher Scientific Italia, Rodano (MI), Italy) filled with filtered sterile oligotrophic seawater amended with f/2 nutrients [45] at 20 °C, 12 h:12 h light:dark cycle, with a photon flux of 100-μmol photons m^−2^ s^−1^ under an agitation trough filtered air influx. Biomass was collected at the stationary growth phase by centrifugation at 3800× *g* at 4 °C for 5 min (Centrifuge 5810 R, Eppendorf, Milan, Italy), immediately frozen in liquid nitrogen and kept at −80 °C until chemical extraction.

### 3.3. Chemical Analysis

#### 3.3.1. Microalgal Pellet Extraction, Extract Fractionation and HPLC Isolation of Pure Metabolites

Frozen wet pellets (4.38 g) of *T. rotula* from 8 l cultures were extracted by ultrasound three times with methanol (1:5, *w:v*) at room temperature. The combined organic phases were filtered, and the solvent was removed under vacuum by rotavapor, affording a raw extract of 342.8 mg.

Fractionation of the methanolic extract was carried out on a CHROMABOND^®^ (Macherey-Nagel) HR-X cartridge (6 mL/500 mg). The cartridge was conditioned with 3 mL of methanol and equilibrated with 6 mL of distilled water. An aliquot of the extract (40 mg) was suspended in 1 mL of distilled water and sonicated for a few seconds in an ultrasonic bath before loading onto the column. After a preliminary desalting step with 3 mL of distilled water, a fractionation of the extract was achieved by elution with 100% H_2_O (fraction A, 18 mL), followed by CH_3_OH/H_2_O (fraction B, 50:50, 18 mL), ACN/H_2_O (fraction C, 70:30, 18 mL), 100% ACN (fraction D, 18 mL) and, finally, CH_2_Cl_2_/MeOH (fraction E, 90:10, 18 mL).

The SPE fractions were analysed by TLC developed with petroleum ether/diethyl ether (60:40, *v/v*), CHCl_3_: MeOH (95:5, *v/v*) and CHCl_3_:MeOH:H_2_O (65:25:4, *v/v/v*) and revealed by spraying with Ce(SO_4_)_2_. Furthermore, for each fraction, a ^1^H NMR spectrum in CD_3_OD or CDCl_3_ and a LC-MS profile on a Kinetex Biphenyl column 2.6 μm, 150 × 2.1 mm, Phenomenex, Castel Maggiore, Bologna, Italy), according to the method reported in [46], were recorded.

HPLC purification of the SPE fractions was carried out on a RP-18 column (Phenomenex, Luna C18(2), 250 × 4.6 mm, 5 µm), with a linear gradient of MeOH/H_2_O from 90% of MeOH to 100% in 10 min and holding for 30 min at 100% MeOH before returning to the initial conditions, flow 1 mL/min monitoring at 210 and 254 nm. Fractions A–M were collected and tested for cytotoxic activity.

#### 3.3.2. GC-MS Analysis

Phytol and 24-MChol isolated from *T. rotula* were analysed by GC-MS using the following temperature gradient: initial 160 °C holding for 3 min; then 5 °C/min up to 260 °C, followed by 30 °C/min up to 310 °C and holding for 3 min at 310 °C; the split flow was 10 mL/min. Full scan *m/z* 50–450. The retention time and mass spectra of the natural compounds were superimposable with those of standard products run in the same experimental conditions.

### 3.4. Biological Assays

#### 3.4.1. Cell Cultures and Treatment

The human breast cancer cell line MCF7 (ATCC HTB-22), the human lung carcinoma cell line A549 (ATCC CCL-185), the human colon adenocarcinoma SW480 (ATCC CCL-185) and the human histiocytic lymphoma U937 (ATCC CRL-1593.2) were purchased from the ATCC cell bank. MePR2B is a primary cell line immortalised from human amniocytes [42]. MCF7 and A549 cells were both grown in DMEM medium (Sigma-Aldrich) supplemented with 10% FBS, 100-U/mL penicillin G, 100-U/mL streptomycin and 2-mM L-glutamine (Lonza, Cologne, Germany). The SW480, U937 and MePR2B cell lines were grown in RPMI 1640 medium, 4.5-g/l glucose (Euroclone, Milan, Italy) supplemented with 10% foetal bovine serum (FBS) (Gibco), 100-U/mL penicillin G, 100-U/mL streptomycin and 2-mM L-glutamine (Lonza, Cologne, Germany). Cells were treated with increasing concentrations of 24-MChol and β-SIT.

#### 3.4.2. MTT Analysis

The MTT (3-(4,5-dimethylthiazol-2-yl)-2, 5-diphenyltetrazolium bromide) assay was used to measure the inhibition of viability following cell treatments with a methanolic extract, fractions and pure compounds **1** and **2** at various concentrations. MTT was added and incubated for four hours at 5% CO_2_ and 37 °C. Four hours later, the formazan precipitate was dissolved in 100 μL of DMSO, and then, the absorbance was measured on an ELISA reader (Thermo Molecular Devices Co., Union City, CA, USA) at 550 nm.

#### 3.4.3. Cell Proliferation Using the Dye Exclusion Test

The cell proliferation assay was performed by the trypan blue dye exclusion test. MCF7, A549 and MePR2B cells were plated into 24-well plates in triplicate and administered the vehicle (75% ethanol + 25% DMSO). Cells were treated for 24 h and 48 h and, afterwards, were diluted in a 1:1 ratio in trypan blue (Sigma-Aldrich, Milan, Italy) and counted with an optical microscope in order to discriminate dead cells (blue) from living cells, which do not stain.

#### 3.4.4. Cell Cycle Analysis

The cell cycle analysis assay was performed using flow cytometry. Treated and untreated MCF7 and A549 MePR2B cells were detached in trypsin-EDTA, washed once with PBS, fixed in iced ethanol 70% and incubated with 50-μg/mL PI (Sigma-Aldrich, Milan, Italy), plus RNase 1 mg/mL for 120 min at 4 °C in the dark. Stained nuclei were analysed with a FACS Canto II (Becton & Dickinson, Mountain View, CA, USA) and the data analysed using ModFit 2.0 cell cycle analysis software (Verity Software House, Topsham, UK). Experiments were repeated three times, with three triplicates for each experiment.

#### 3.4.5. Apoptosis Evaluation

Caspase-3 activity was evaluated in living cells using CaspGLOW fluorescein active caspase-3, according to the manufacturer’s instructions. Briefly, A549 and MePR2B cells were treated with 1 µL of FITC-DEVD-FMK for 60 min in a 37 °C incubator with 5% CO_2_ compared to the untreated cells. After this time, the cells were detached and analysed with FACS Canto II (Becton & Dickinson, Mountain View, CA, USA). The data were evaluated using DIVA Software version 8.0. Since caspase-3 is not expressed in MCF7 cells, apoptosis and caspase-9 evaluation was performed. Apoptosis was analysed using the FITC Annexin V Apoptosis Detection Kit (BD Pharmingen) according to the manufacturer’s instructions. Briefly, the cells were incubated with 5 µL of PI and 5 µL of Annexin V FITC in agitation for 15 min at room temperature. After this time, the stained cells were analysed using the FACS Canto II. Apoptotic cell death was assessed by counting the number of cells that stained positive for Annexin V–FITC and negative for propidium iodide. The data were analysed using DIVA Software version 8.0 (Becton & Dickinson, San Jose, CA, USA). The caspase-9 evaluation was performed using the SR-FLICA^®^ Caspase-9 Assay Kit according to the manufacturer’s instructions. Briefly, after the treatments, the cells were collected, incubated at room temperature in the dark with the SR-FLICA^®^ reagent (1× from 150× accordingly with the product data sheet) and subsequently analysed with FACS Canto II. The data were evaluated using DIVA Software version 8.0.

#### 3.4.6. Western Blot Analysis

For Western blot detection, the cells were washed with cold 1× PBS and lysed using a lysis buffer containing 50-mM Tris-HCl, pH 7.4, 150-mM NaCl, 1% NP40, 10-mM NaF, 1-mM PMSF (phenylmethylsulphonyl fluoride) and a protease inhibitor cocktail (Roche). The cells were then centrifuged at 13,000 rpm for 15 min at 4 °C, and the protein content of the supernatant was used to determine the protein concentration by the colorimetric assay (Bio-Rad, Italy). Cell extracts were diluted 1:1 in the sample buffer 2× Laemmli (0.217-M Tris-HCl, pH 8.0, 52.17% SDS, 17.4% glycerol, 0.026% bromophenol blue and 8.7% β-mercaptoethanol) and then boiled for 3 min. Equal amounts of protein (50 μg) were run and separated by SDS-PAGE gel (acrylamide gel). The primary antibodies used were N-cadherin (ELABSCIENCE, E-AB-70061), FAS (ELABSCIENCE, E-AB-60029) and TRAIL (Abcam, ab2056). Alpha-tubulin (ELABSCIENCE, E-AB-20036) antibodies was used for normalisation.

#### 3.4.7. Real-Time Cell Proliferation Assay

Cell proliferation was monitored with the xCELLigence system (Roche, Mannheim, Germany). A549, MCF7, SW480, U937 and MePR2B cells were suspended into the corresponding culture medium and added to a 96-well microtiter plate that is specifically designed to measure cellular impedance (E-Plate, Roche, Mannheim, Germany). Following cell adhesion (4 h), cells were treated with increasing concentrations (5–50 μM) of 24-MChol. The measured impedance, which is dependent on the level of confluence (20,000 cells/well), was expressed as an arbitrary unit called the Cell Index (CI). Dynamic CI values were monitored at 15-min intervals from the time of plating until the end of the experiment. CI values were calculated and plotted on the graph. Standard deviations of triplicate wells for each cell type with different treatments were analysed using RTCA software (Roche, Mannheim, Germany).

#### 3.4.8. Statistical Analysis

Experiments were carried out at least in triplicate, and the values were expressed as the mean ± SD. The comparative statistical analyses were done with the Student’s *t*-test and one-way ANOVA using GraphPad Prism 5.01 (GraphPad Software, Inc., San Diego, CA, USA). *p*-values < 0.05 were considered statistically significant (a = *p* ≤ 0.05, b = *p* ≤ 0.01, c = *p* ≤ 0.001 and d = *p* ≤ 0.0001). The IC_50_ was determined for subfractions of *T. rotula* with respect to the relative control on the MCF7 cell line (Supplementary Figure S1). IC_50_ values were calculated using a four-parameters logistic curve with an IC = 95%. The goodness-of-fit curves were quantified with the least squares fit method and R^2^ value calculations.

## 4. Conclusions

In this study, 24-MChol was investigated for the first time for its potential applications in the prevention and treatment of cancer. In vitro studies were carried out in parallel with the most studied β-SIT on two model cancer systems: the human breast MCF7 cell line, already investigated for β-SIT treatments, and the human lung A549 tumour cell line. Our data showed for the first time that 24-MChol induces cytotoxic and antiproliferative effects in the µM range with a high sensitivity for both cell lines. 24-MChol promotes cell death through apoptosis, as revealed by cell cycle analysis and confirmed by the increase in caspase-3 in A549 and the presence of annexin-positive cells, as well as caspase-9 activation in the MCF7 cell line. These results are further corroborated by the increase in the FAS protein in both cell lines. Noteworthy, we showed that 24-MChol induces the activation of different cell death pathways. The expression of TRAIL increased only in MCF7 cells, which were more marked following the treatment with 24-MChol at 3 and 30 μM, and it also seemed to have different effects compared to β-SIT. A deeper understanding of the mechanisms activated and the possible involvement of other intriguing and less investigated targets, such as the sphingomyelin cycle, will be crucial to assessing the potential of this compound for cancer treatment and prevention. The possibility to obtain the product by biotechnological approaches exploiting its microalgal origin and abundance represents an attractive perspective that may stimulate further studies, particularly in view of the potential use of *T. rotula* in formulations for the development of nutraceutical products.

## Supplementary Materials

The following supporting information can be downloaded at https://www.mdpi.com/article/10.3390/md20100595/s1: Figure S1. IC_50_ assessment of *Thalassiosira rotula* HPLC subfractions (B, D, F, H and L) on the MCF7 cell line after 24-h exposure. Figure S2. Cell viability (MTT assay) of the MCF7 cell line after 48-h exposure to the *Thalassiosira rotula* methanolic extract (EXT), SPE fractions (A–E) and HPLC peaks (B, D, F, H and L). Figure S3. ^1^H-NMR spectrum (600 MHz, CDCl_3_) of HPLC peak D (fucoxanthin). Figure S4. HR-ESI MS (upper) and MS/MS (lower) spectrum of HPLC peak D (fucoxanthin). Figure S5. ^1^H-NMR spectrum (600 MHz, CDCl_3_) of HPLC peak F (phytol). Figure S6. EI-MS spectrum of HPLC peak F (phytol). Figure S7. ^1^H-NMR spectrum (600 MHz, CDCl_3_) of HPLC peak H. Figure S8. ^1^H-NMR spectrum (600 MHz, CDCl_3_) of HPLC peak L (24-methylene cholesterol). Figure S9. EI-MS spectrum of HPLC peak L (24-methylene cholesterol). Figure S10. Dynamic monitoring of A549 cell proliferation. A549 cells were monitored in a 96-well E-plate at a density of 20,000 cells/well. Four hours after seeding, the cells were treated with increasing concentrations of 24-methylene cholesterol (5–50 μM) for 48 h. Data shown are the mean ± SEM of at least three independent experiments performed in triplicate. Figure S11. Dynamic monitoring of MCF7 cell proliferation. MCF7 cells were monitored in a 96-well E-plate at a density of 20,000 cells/well. Four hours after seeding, the cells were treated with increasing concentrations of 24-methylene cholesterol (5–50 μM) for 48 h. Data shown are the mean ± SEM of at least three independent experiments performed in triplicate. Figure S12. Dynamic monitoring of SW480 cell proliferation. SW480 cells were monitored in a 96-well E-plate at a density of 20,000 cells/well. Four hours after seeding, the cells were treated with increasing concentrations of 24-methylene-cholesterol (5–50 μM) for 48 h. Data shown are the mean ± SEM of at least three independent experiments performed in triplicate. Figure S13. The cell proliferation rates on U-937 cells after 24 and 48 h of treatment with 24-methylene cholesterol at increasing concentrations (5–30 μM). Data shown are the mean ± SEM of at least three independent experiments performed in triplicate. Figure S14. Dynamic monitoring of MePR2B cell proliferation. MePR2B cells were monitored in a 96-well E-plate at a density of 20,000 cells/well. Four hours after seeding, the cells were treated with increasing concentrations of 24-methylene cholesterol (5–50 μM) for 48 h. Data shown are the mean ± SEM of at least three independent experiments performed in triplicate. Figure S15. Cell proliferation rates in the (a) A549, (b) MCF7 and (c) MePR2B cell lines after treatment with β-SIT or 24-MChol at concentrations of 0.3, 3 and 30 μM observed after 48 h. Figure S16. Cell viability curves (MTT) of (a) the A549, (b) MCF7 and (c) MePR-2B cell lines after 24 h of treatment with β-SIT or 24-MChol at the concentrations of 0.3, 3 and 30 μM (CTR, no treatment; 0.5% EtOH/DMSO, dissolving solvent). Figure S17. DNA content flow cytometry histograms about the cell cycle analysis after the treatment of A549, MCF7 and MePR2B cells with 24-MChol and β-SIT (0.3–3–30 μM) for 24 h. Figure S18. Apoptosis flow cytometry analysis of MCF7 cells. Annexin V/PI staining with the FITC Annexin V Apoptosis Detection Kit in MCF7 cells treated with 24-MChol and β-SIT (0.3–30 μM) for 24 h. Figure S19. Original blots (biological replicates) for FAS in A549 after treatments with β-SIT and 24-MChol at increasing concentrations (0.3–30 μM). Figure S20. Original blots (biological replicates) for FAS in MCF7 after treatments with β-SIT and 24-MChol at increasing concentrations (0.3–30 μM). Figure S21. Original blots (biological replicates) for TRAIL in A549 after treatments with β-SIT and 24-MChol at increasing concentrations (0.3–30 μM). Figure S22. Original blots (biological replicates) for TRAIL in MCF7 after treatments with β-SIT and 24-MChol at increasing concentrations (0.3–30 μM).
