# Supplementary material for: Cytotoxic Potential of the Marine Diatom Thalassiosira rotula: Insights into Bioactivity of 24-Methylene Cholesterol
Source: Mar Drugs. 2022 Sep 23;20(10):595. doi: 10.3390/md20100595 (PMC9604713; doi:10.3390/md20100595)

marine drugs

Supplementary material

Cytotoxic potential of the marine diatom *Thalassiosira rotula*: insights into bioactivity of 24-methylene-cholesterol

Adele Cutignano [1,2,†,*], Mariarosaria Conte [3,†], Virginia Tirino [4], Vitale Del Vecchio [4], Roberto De Angelis [2], Angela Nebbioso [3], Lucia Altucci [3,5#] and Giovanna Romano [2#]

1 CNR-Institute of Biomolecular Chemistry-Via Campi Flegrei, 34-80078 Pozzuoli (Napoli), Italy;
2 Stazione Zoologica Anton Dohrn, Ecosustainable Marine Biotechnology Department, Villa Comunale, 80121 Napoli, Italy
3 Department of Precision Medicine, University of Campania 'L. Vanvitelli', Via L. De Crecchio 7, 80138 Napoli, Italy
4 Department of Experimental Medicine, Section of Biotechnology, Molecular Medicine and Medical Histology, University of Campania "L. Vanvitelli", Via L. de Crecchio 7, 80138 Napoli, Italy
5 Biogem, Institute of Molecular Biology and Genetics, Via Camporeale Area P.I.P., 83031 Ariano Irpino (Avellino) Italy.
* Correspondence: acutignano@icb.cnr.it; Tel.: +39 081 8675313
† These authors equally contributed to this work
# These authors equally contributed to this work

List of Figures.

Figure S1. IC_{50} assessment of *Thalassiosira rotula* HPLC subfractions (B, D, F, H, L) on MCF7 cell line after 24 h exposure.

Figure S2. Cell viability (MTT assay) of MCF7 cell line after 48 h exposure to *Thalassiosira rotula* methanolic extract (EXT), SPE-fractions (A-E) and HPLC peaks (B, D, F, H, L).

Figure S3. ^{1}H-NMR spectrum (600MHz, $CDCl_3$) of HPLC peak D (fucoxanthin).

Figure S4. HR-ESI MS (upper) and MS/MS (lower) spectrum of HPLC peak D (fucoxanthin).

Figure S5. ^{1}H-NMR spectrum (600MHz, $CDCl_3$) of HPLC peak F (phytol).

Figure S6. EI-MS spectrum of HPLC peak F (phytol).

Figure S7. ^{1}H-NMR spectrum (600MHz, $CDCl_3$) of HPLC peak H.

Figure S8. ^{1}H-NMR spectrum (600MHz, $CDCl_3$) of HPLC peak L (24-methylene cholesterol).

Figure S9. EI-MS spectrum of HPLC peak L (24-methylene cholesterol).

Figure S10. Dynamic monitoring of A549 cell proliferation. A549 cells were monitored in 96-well E-plate at the density of 20000 cells/well. Four hours after seeding, cells were treated with increasing concentrations of 24-methylene cholesterol (5-50 μM) for 48 h. Data shown are the mean ± SEM of at least three independent experiments performed in triplicate.

Figure S11. Dynamic monitoring of MCF7 cell proliferation. MCF7 cells were monitored in 96-well E-plate at the density of 20000 cells/well. Four hours after seeding, cells were treated with increasing concentrations of 24-methylene cholesterol (5-50 μM) for 48 h. Data shown are the mean ± SEM of at least three independent experiments performed in triplicate.

Figure S12. Dynamic monitoring of SW480 cell proliferation. SW480 cells were monitored in 96-well E-plate at the density of 20000 cells/well. Four hours after seeding, cells were treated with increasing concentrations of 24-methylene-cholesterol (5-50 μM) for 48 h. Data shown are the mean ± SEM of at least three independent experiments performed in triplicate.

Figure S13. Cell proliferation rate on U-937cells after 24 and 48 h of treatment with 24-methylene cholesterol at increasing concentrations (5-30 μM). Data shown are the mean ± SEM of at least three independent experiments performed in triplicate.

Figure S14. Dynamic monitoring of MePR2B cell proliferation. MePR2B cells were monitored in 96-well E-plate at the density of 20000 cells/well. Four hours after seeding, cells were treated with increasing concentrations of 24-methylene cholesterol (5-50 μM) for 48 h. Data shown are the mean ± SEM of at least three independent experiments performed in triplicate.

Figure S15. Cell proliferation rates in a) A549, b) MCF7 and c) MePR2Bcell lines after treatment with β-SIT or 24-MChol at concentration 0.3, 3 and 30 μM, observed after 48 h.

Figure S16. Cell viability curves (MTT) of a) A549, b) MCF7 and c) MePR-2B cell lines after 24 h of treatment with β-SIT or 24-MChol at the concentration of 0.3, 3 and 30 μM (CTR, no treatment; 0.5% EtOH/DMSO, dissolving-solvent).

Figure S17. DNA-content flow cytometry histograms about cell cycle analysis after treatment of A549, MCF7 and MePR2B cells with 24-MChol and β-SIT (0.3-3-30 μM) for 24 h.

Figure S18. Apoptosis flow cytometry analysis of MCF7 cells. Annexin V/PI staining with FITC Annexin V Apoptosis Detection Kit in MCF7 cells treated with 24-MChol and β-SIT (0.3-3-30 μM) for 24 h.

Figure S19. Original blots (biological triplicate) for FAS in A549 after treatment with β-SIT and 24-MChol at increasing concentrations (0.3-30 μM).

Figure S20. Original blots (biological triplicate) for FAS in MCF7 after treatment with β-SIT and 24-MChol at increasing concentrations (0.3-30 μM).

Figure S21. Original blots (biological triplicate) for TRAIL in A549 after treatment with β-SIT and 24-MChol at increasing concentrations (0.3-30 μM).

Figure S22. Original blots (biological triplicate) for TRAIL in MCF7 after treatment with β-SIT and 24-MChol at increasing concentrations (0.3-30 μM).

Figure S1. IC_{50} assessment of *Thalassiosira rotula* HPLC subfractions (B, D, F, H, L) on MCF7 cell line after 24 h exposure.

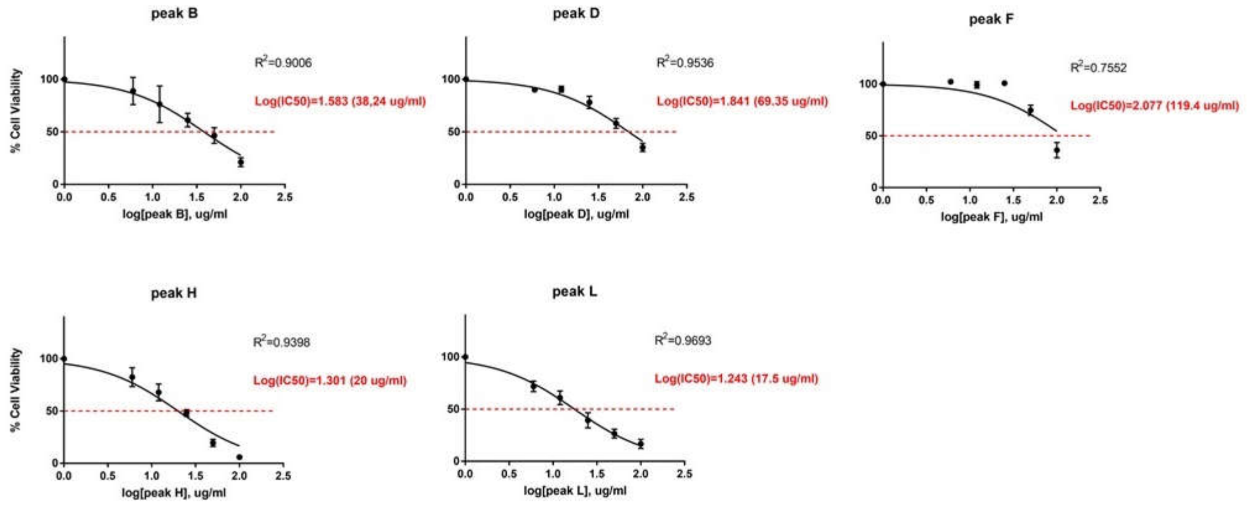

Figure S2. Cell viability (MTT assay) of MCF7 cell line after 48 h exposure to *Thalassiosira rotula* methanolic extract (EXT), SPE-fractions (A-E) and HPLC peaks (B, D, F, H, L). Statistical notations: a, $p \leq 0.05$; b, $p \leq 0.01$; c, $p \leq 0.001$; d, $p \leq 0.0001$.

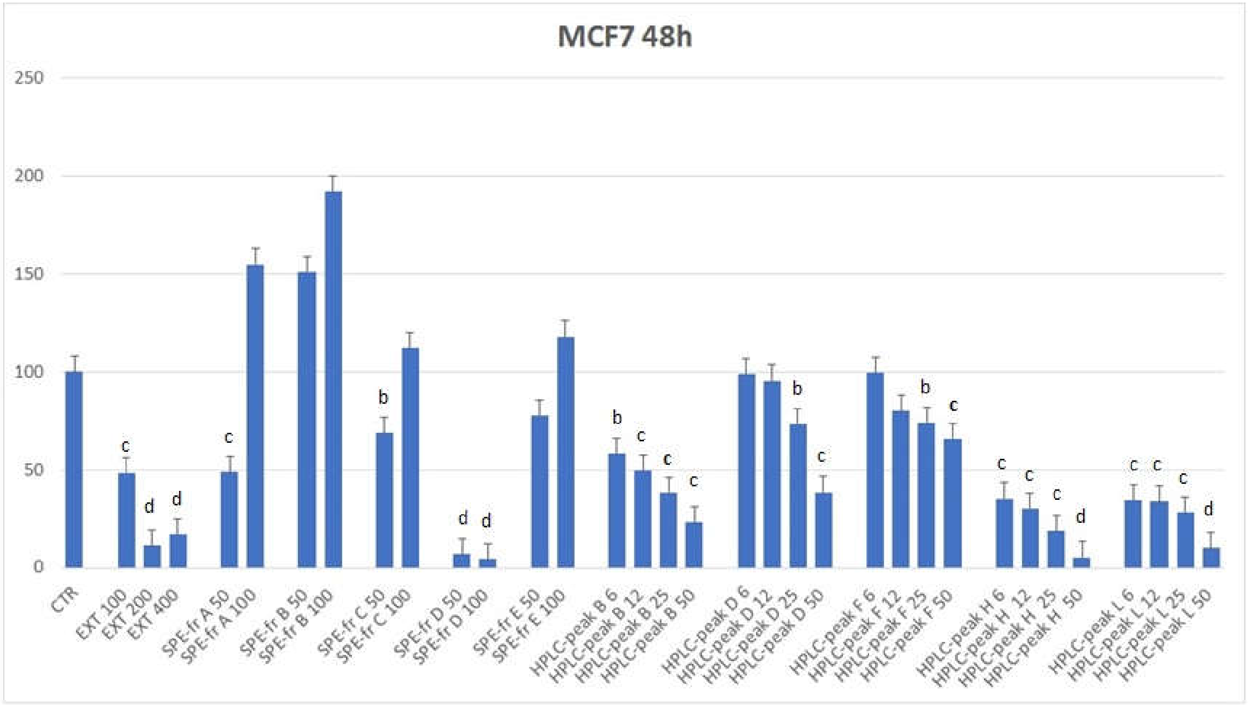

Figure S3. ^{1}H-NMR spectrum (600MHz, $CDCl_3$) of HPLC peak D (fucoxanthin).

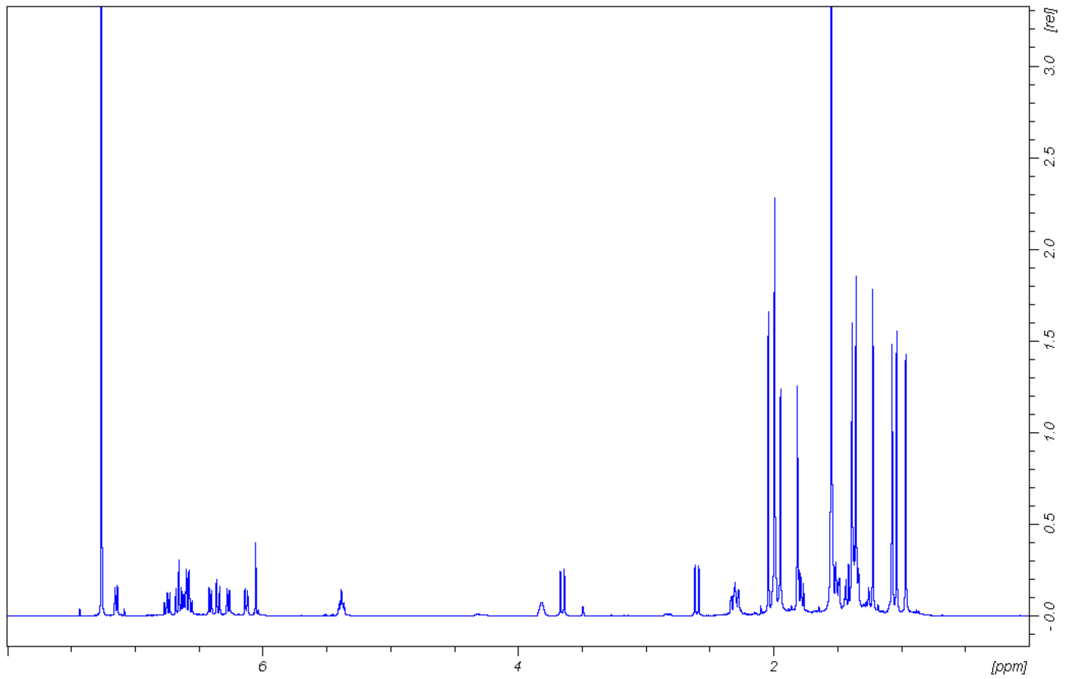

Figure S4. HR-ESI MS (upper) and MS/MS (lower) spectrum of HPLC peak D (fucoxanthin).

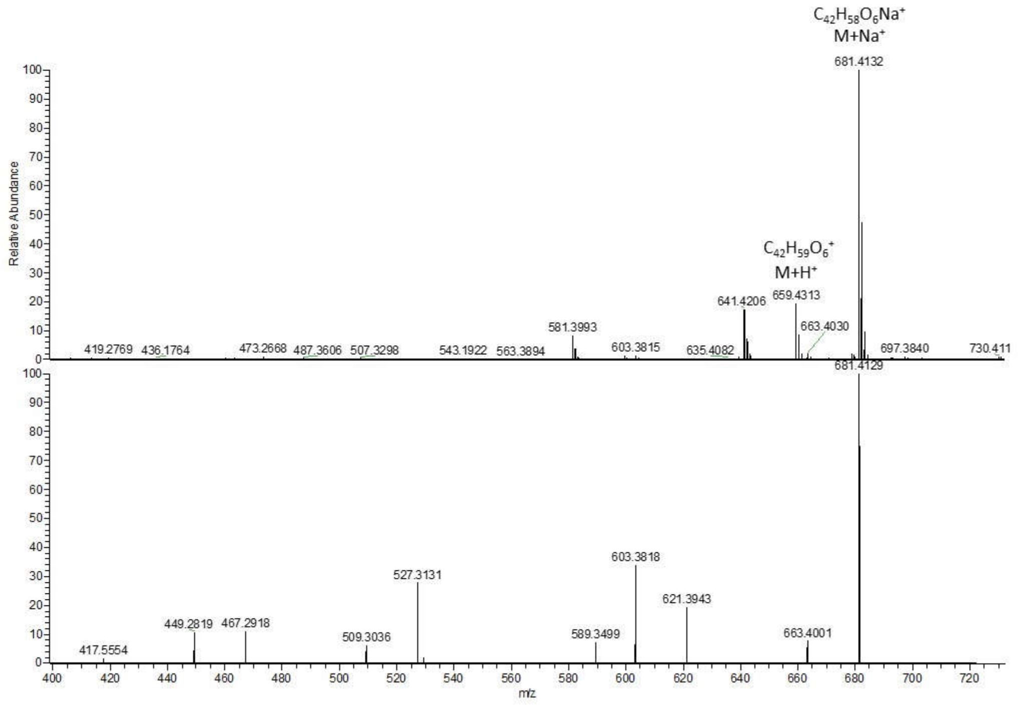

Figure S5. ^{1}H-NMR spectrum (600MHz, $CDCl_3$) of HPLC peak F (phytol).

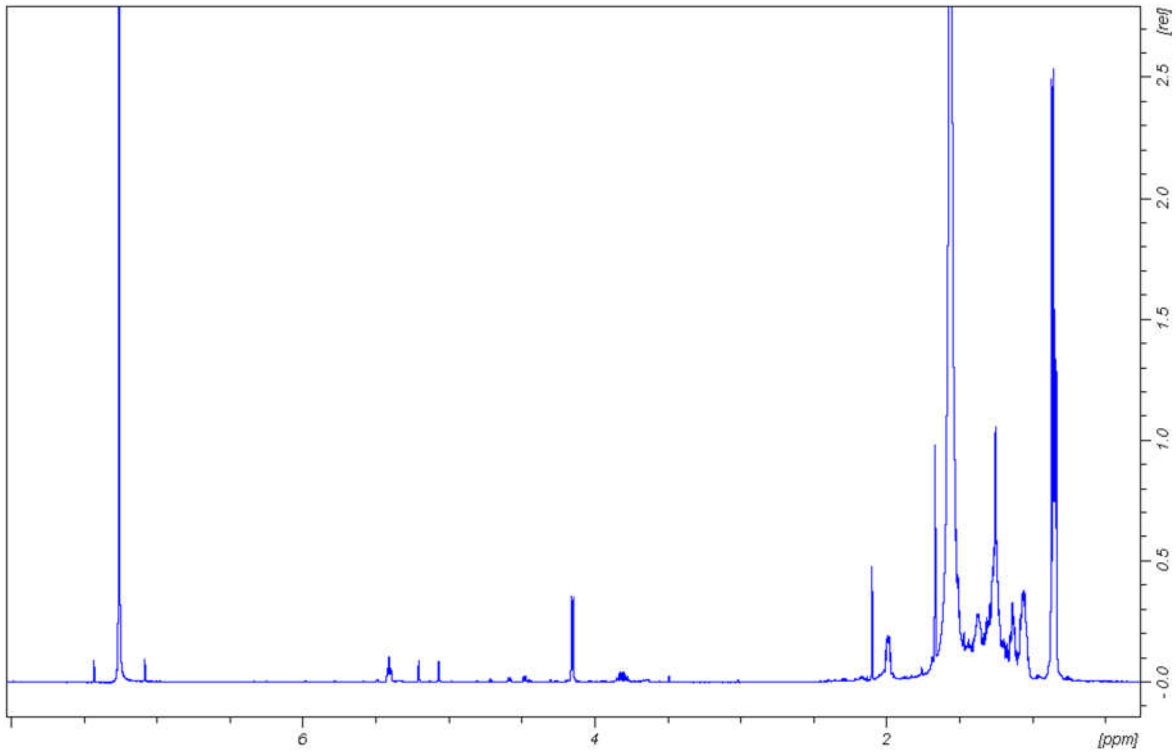

Figure S6. EI-MS spectrum of HPLC peak F (phytol).

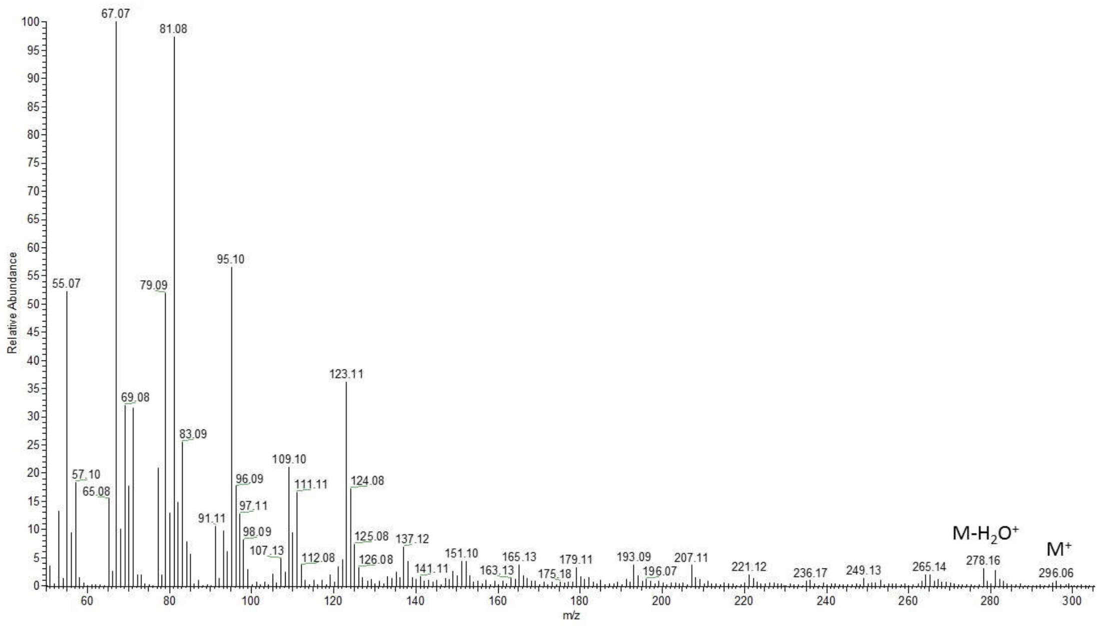

Figure S7. ^{1}H-NMR spectrum (600MHz, $CDCl_3$) of HPLC peak H.

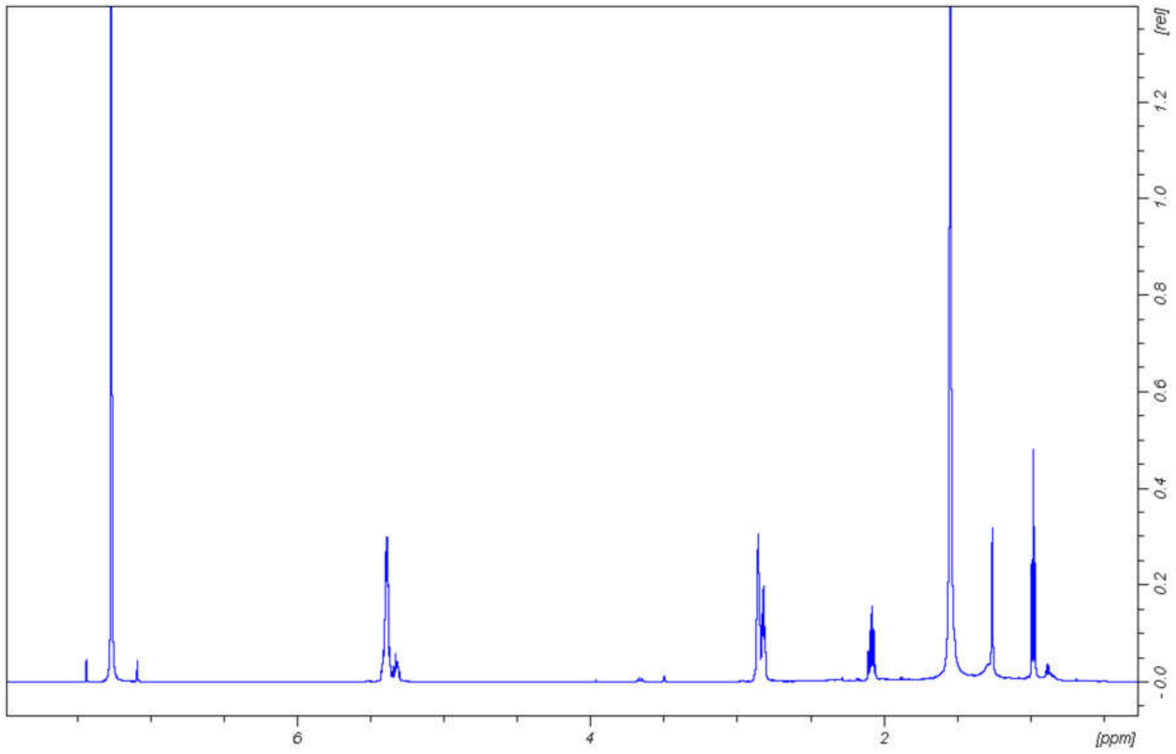

Figure S8. ^{1}H-NMR spectrum (600MHz, $CDCl_3$) of HPLC peak L (24-methylene cholesterol).

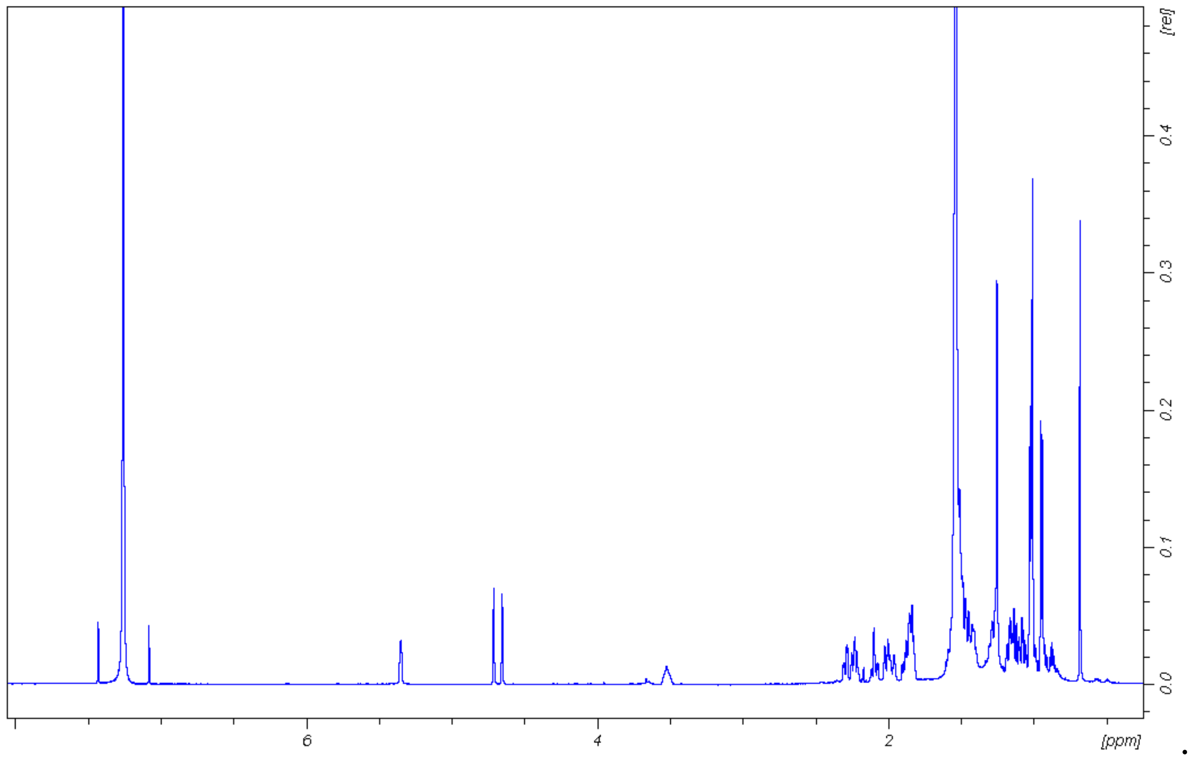

.

Figure S9. EI-MS spectrum of HPLC peak L (24-methylene cholesterol).

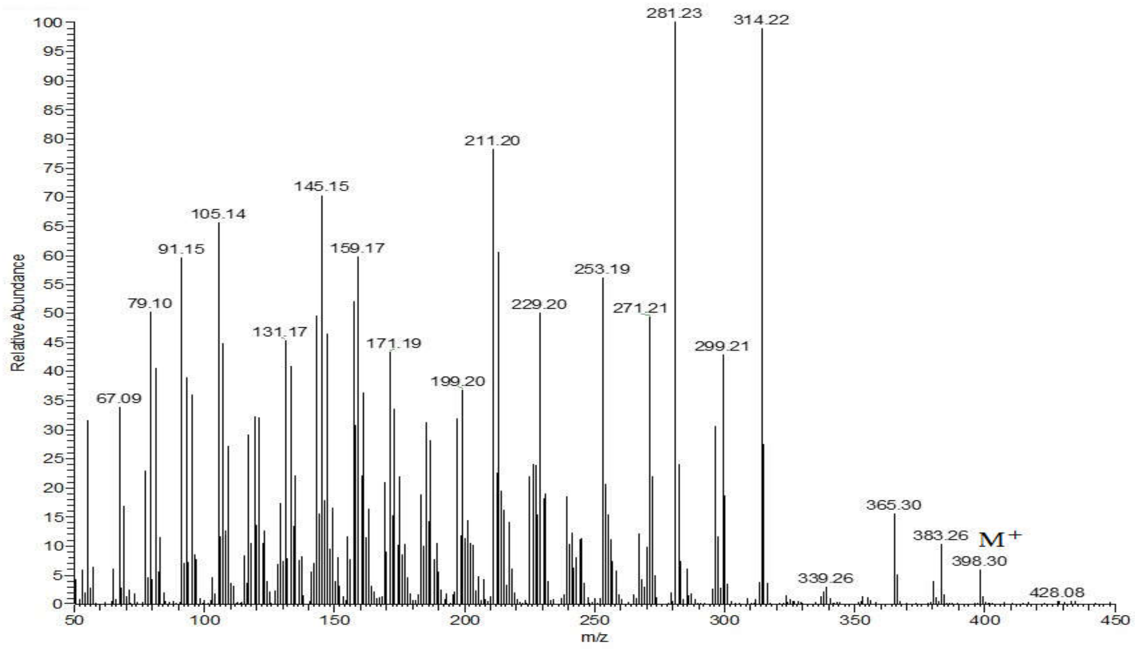

Figure S10: **Dynamic monitoring of A549 cell proliferation.** A549 cells were monitored in 96-well E-plate at the density of 20000 cells/well. Four hours after seeding, cells were treated with increasing concentrations of 24-methylene cholesterol (5-50 µM) for 48 h. Data shown are the mean ± SEM of at least three independent experiments performed in triplicate.

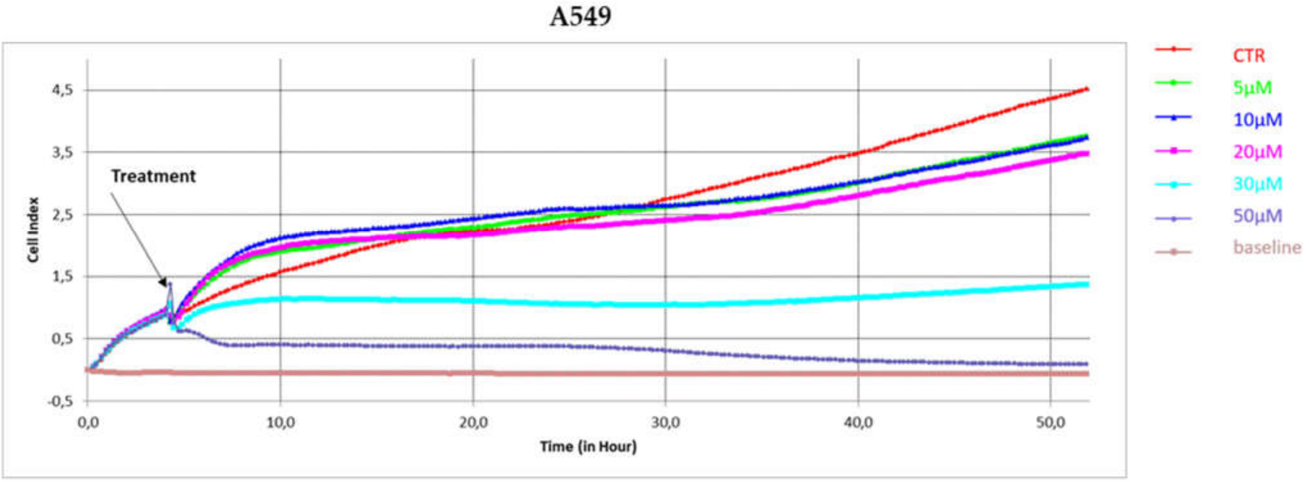

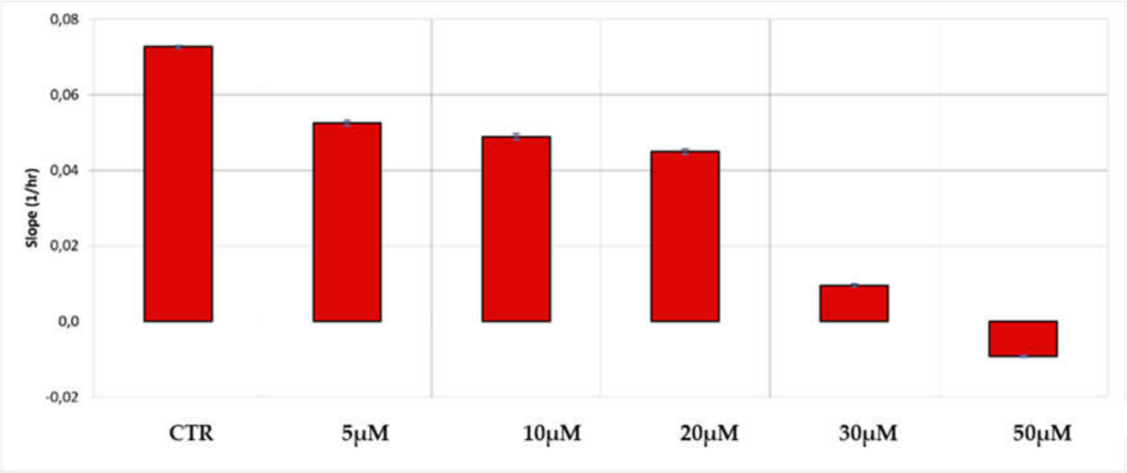

Figure S11: **Dynamic monitoring of MCF7 cell proliferation.** MCF7 cells were monitored in 96-well E-plate at the density of 20000 cells/well. Four hours after seeding, cells were treated with increasing concentrations of 24-methylene cholesterol (5-50 µM) for 48 h. Data shown are the mean ± SEM of at least three independent experiments performed in triplicate.

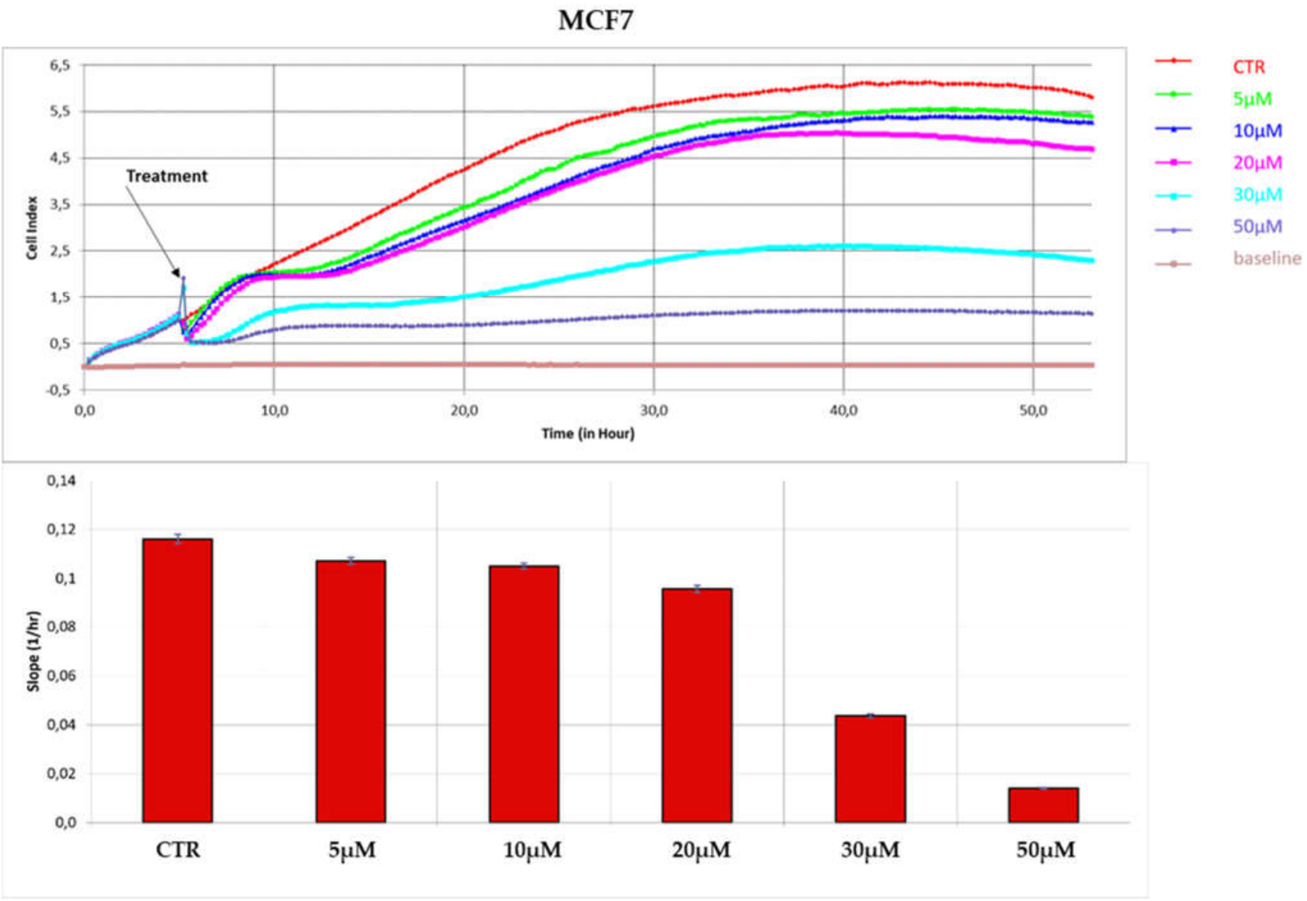

Figure S12: Dynamic monitoring of SW480 cell proliferation. SW480 cells were monitored in 96-well E-plate at the density of 20000 cells/well. Four hours after seeding, cells were treated with increasing concentrations of 24-methylene cholesterol (5-50 μM) for 48 h. Data shown are the mean ± SEM of at least three independent experiments performed in triplicate.

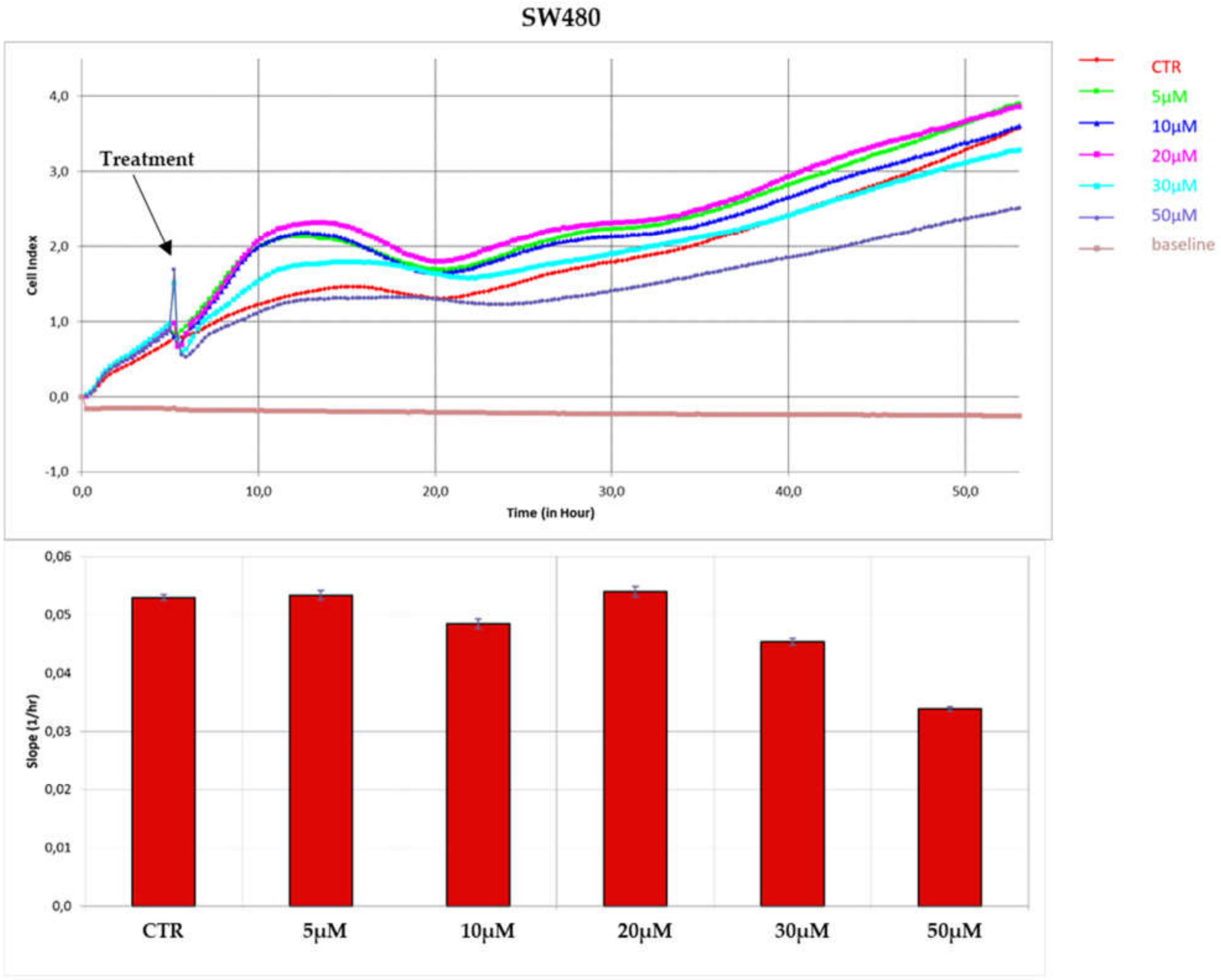

Figure S13: Cell proliferation rate on U-937cells after 24 and 48 h of treatment with 24-methylene cholesterol at increasing concentrations (5-30 μM). Data shown are the mean ± SEM of at least three independent experiments performed in triplicate.

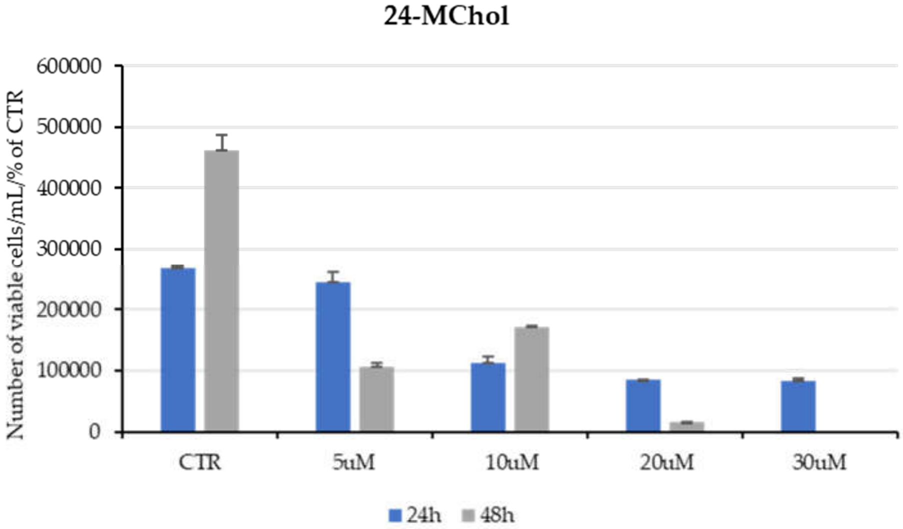

Figure S14: **Dynamic monitoring of MePR2B cell proliferation**. MePR2B cells were monitored in 96-well E-plate at the density of 20000 cells/well. Four hours after seeding, cells were treated with increasing concentrations of 24-methylene cholesterol (5-50 μM) for 48 h. Data shown are the mean ± SEM of at least three independent experiments performed in triplicate.

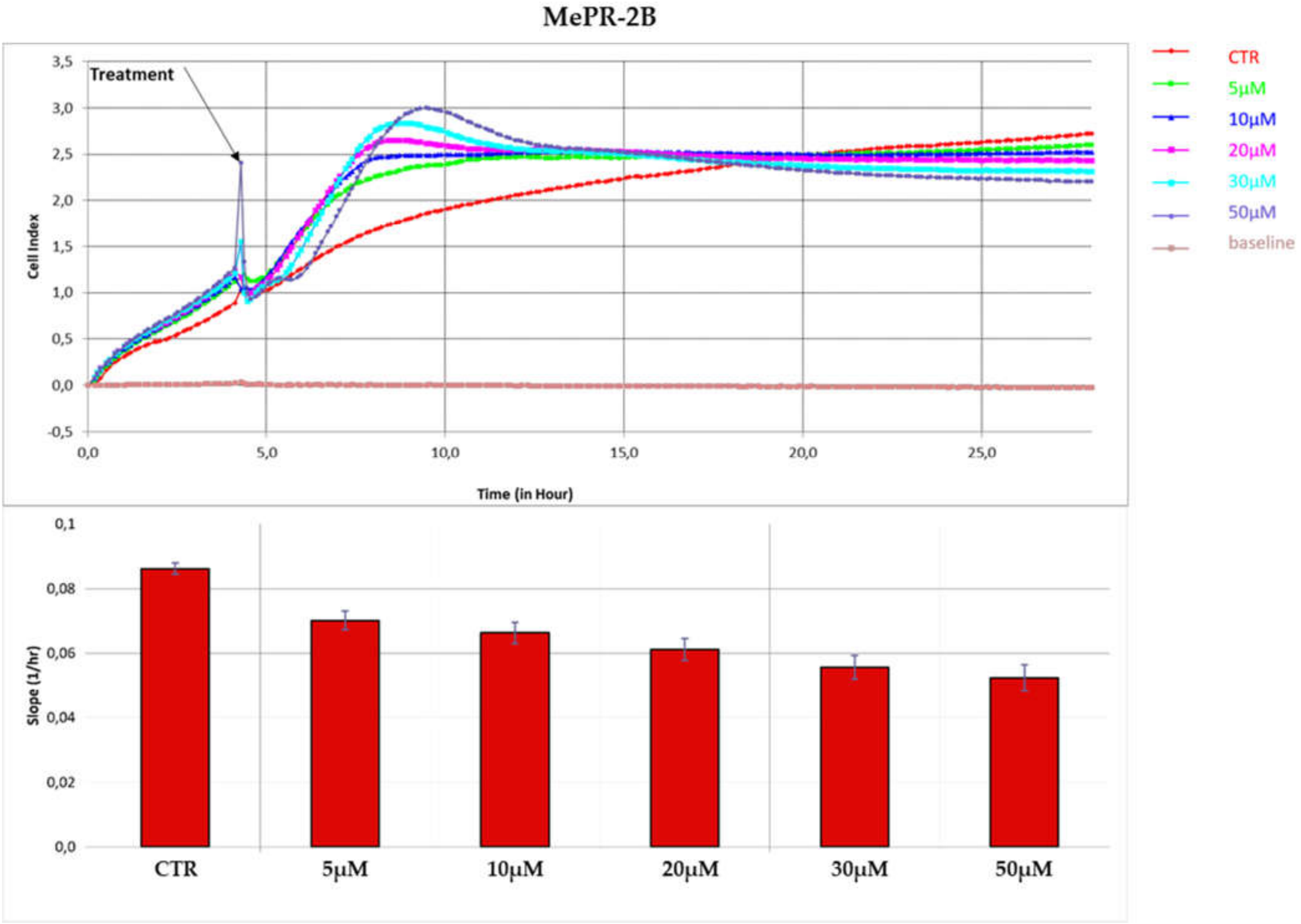

Figure S15. Cell proliferation rates in a) A549, b) MCF7 and c) MePR2B cell lines after treatment with β-SIT or 24-MChol at concentration 0.3, 3 and 30 μM, observed after 48 h. Statistical notations: a, $p \leq 0.05$; b, $p \leq 0.01$; c, $p \leq 0.001$; d, $p \leq 0.0001$.

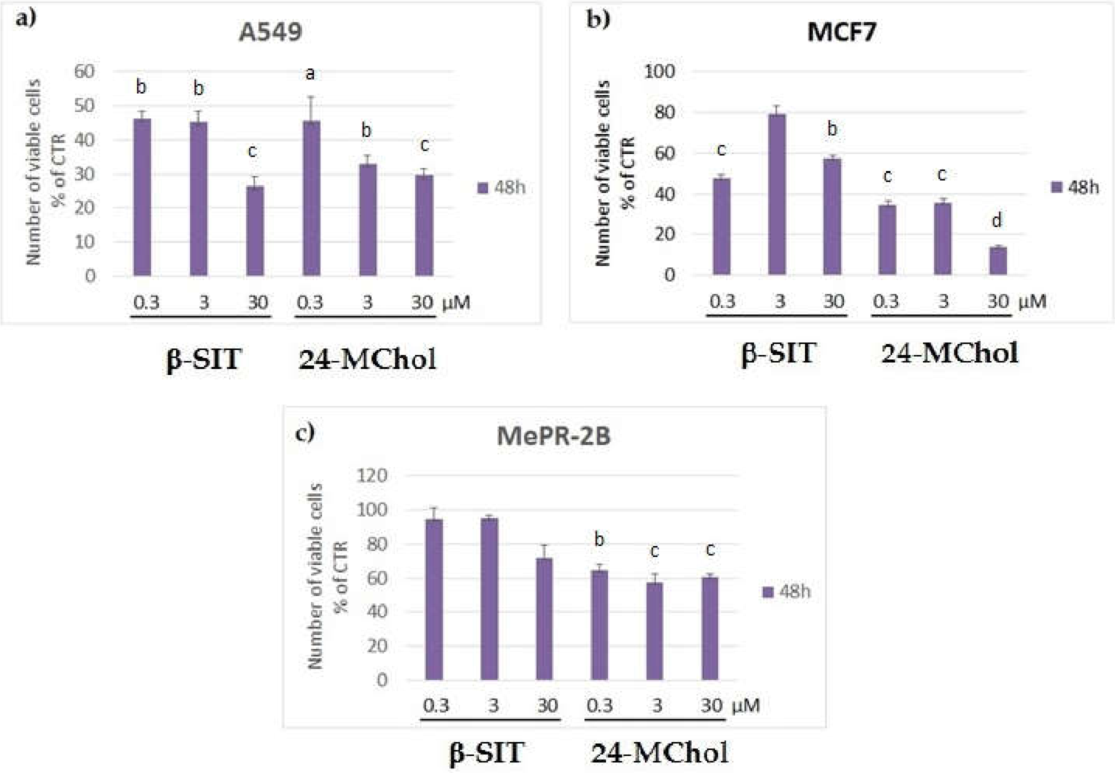

Figure S16. Cell viability curves (MTT) of a) A549, b) MCF7 and c) MePR-2B cell lines after 24 h of treatment with β-SIT or 24-MChol at the concentration of 0.3, 3 and 30 μM (CTR, no treatment; 0.5% EtOH/DMSO, dissolving-solvent).

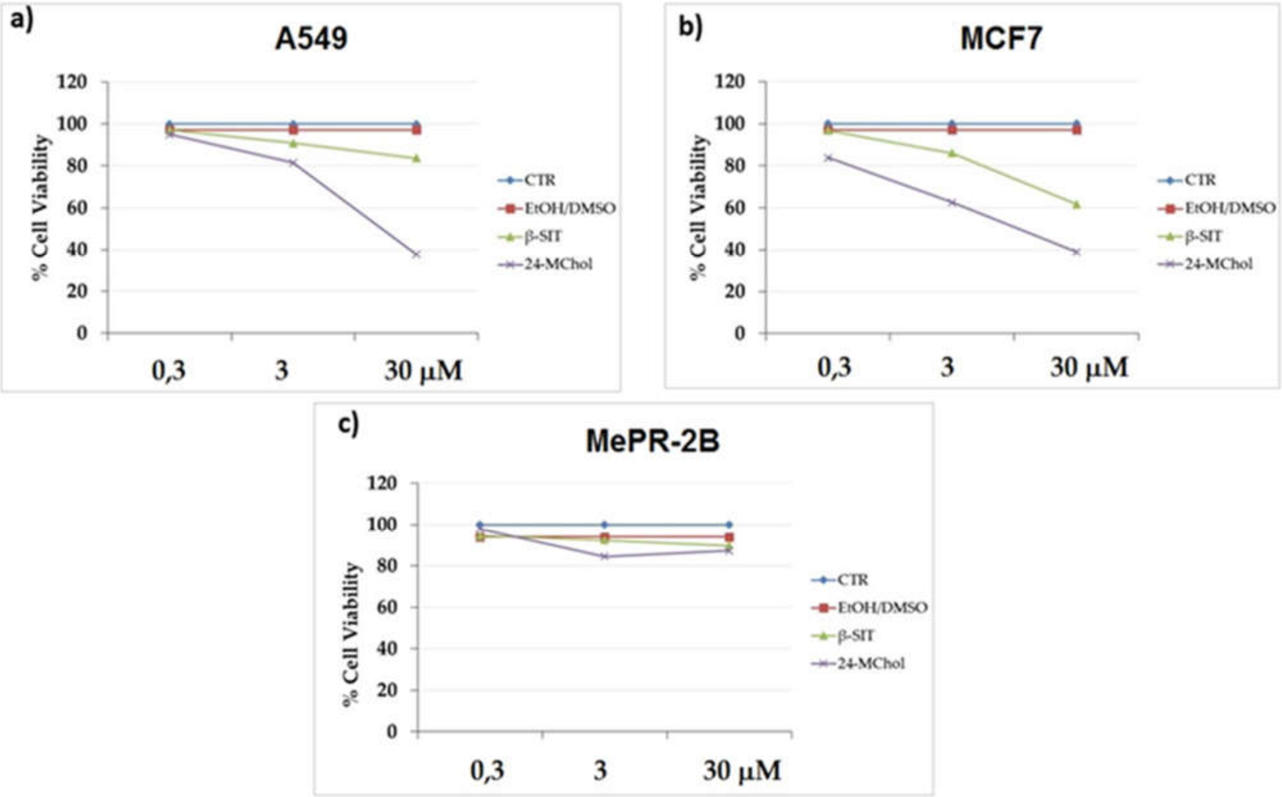

Figure S17. DNA-content flow cytometry histograms about cell cycle analysis after treatment of A549, MCF7 and MePR2B cells with 24-methylene cholesterol and β-Sitosterol (0.3-3-30 μM) for 24 h.

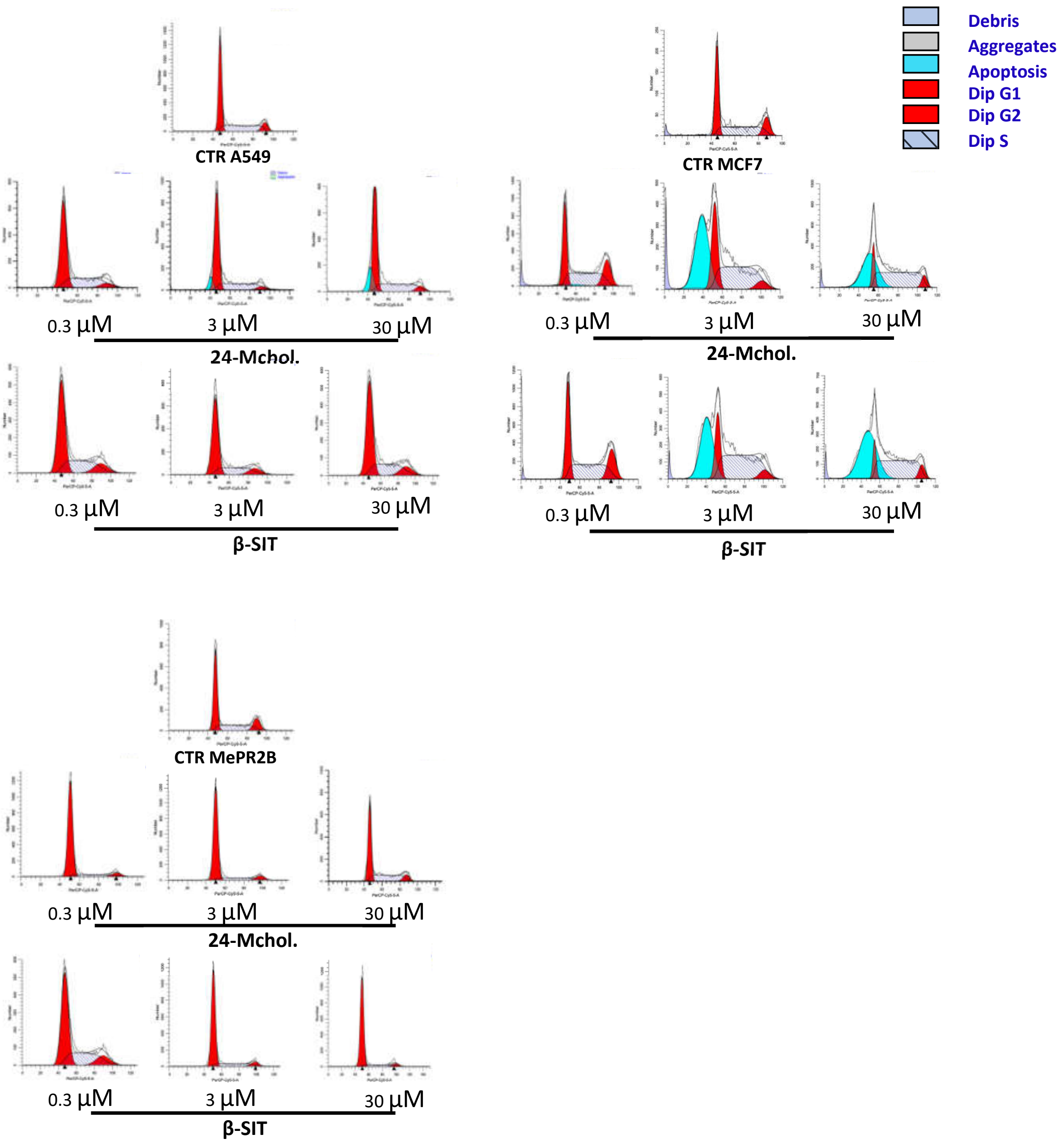

Figure S18: Apoptosis flow cytometry analysis of MCF7 cells. Annexin V/PI staining with FITC Annexin V Apoptosis Detection Kit in MCF7 cells treated with 24-methylene cholesterol and β-Sitosterol (0.3-3-30 μM) for 24 h.

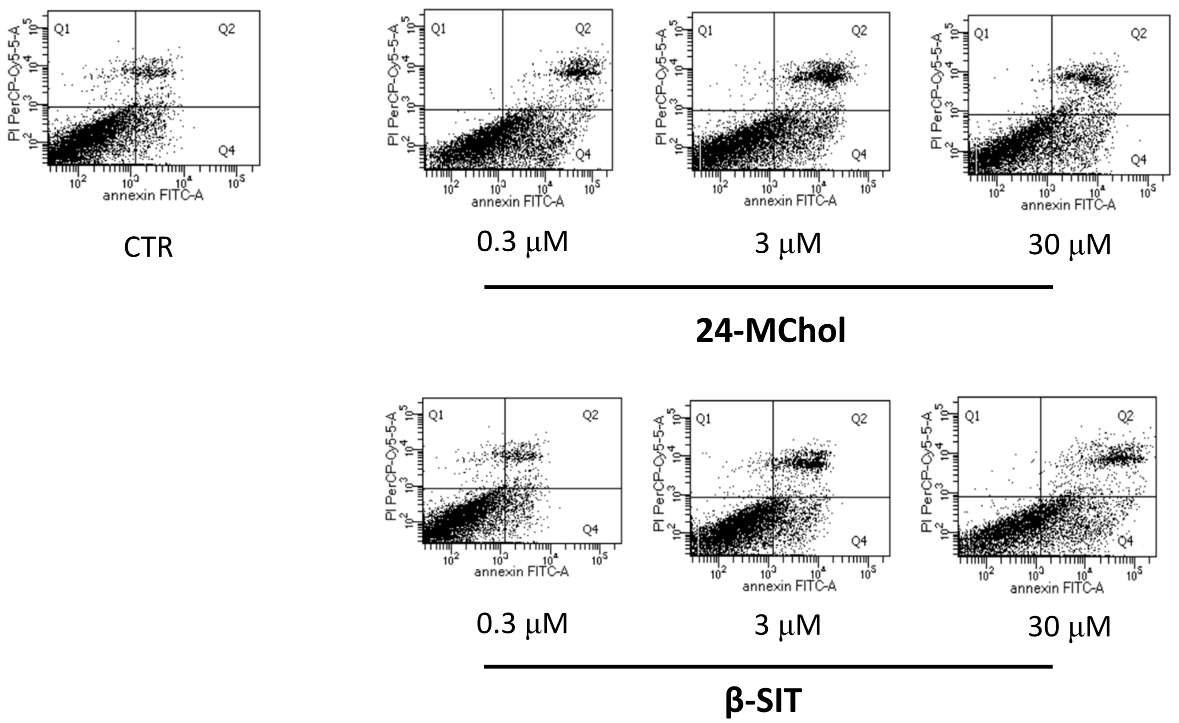

Figure S19. Original blots (biological triplicate) for FAS (upper panels) in A549 after treatment with β-SIT and 24-MChol at increasing concentrations (0.3-30 μM). Alpha tubulin (lower panels) has been used as loading control. The arrows indicate the reference band corresponding to the marker with the molecular weights used during migration. The red dial indicates the band inserted into the manuscript

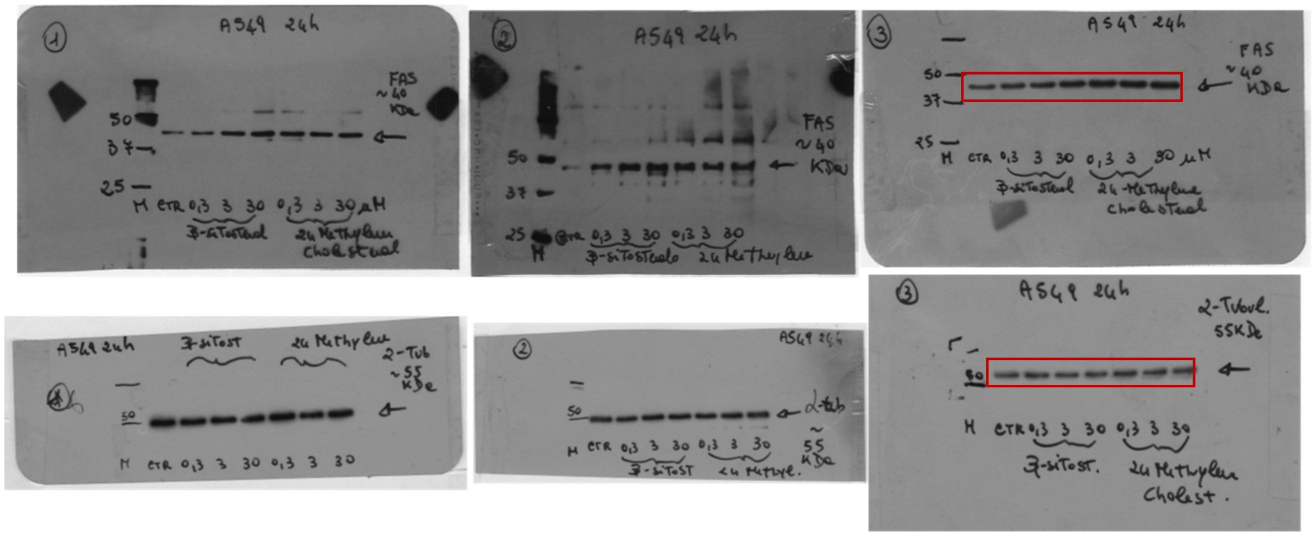

Figure S20. Original blots (biological triplicate) for FAS (Upper panel) in MCF7 after treatment with β-SIT and 24-MChol at increasing concentrations (0.3-30 μM). Alpha tubulin (lower panel) has been used as loading control. The arrows indicate the reference band corresponding to the marker with the molecular weights used during migration. The red dial indicates the band inserted into the manuscript

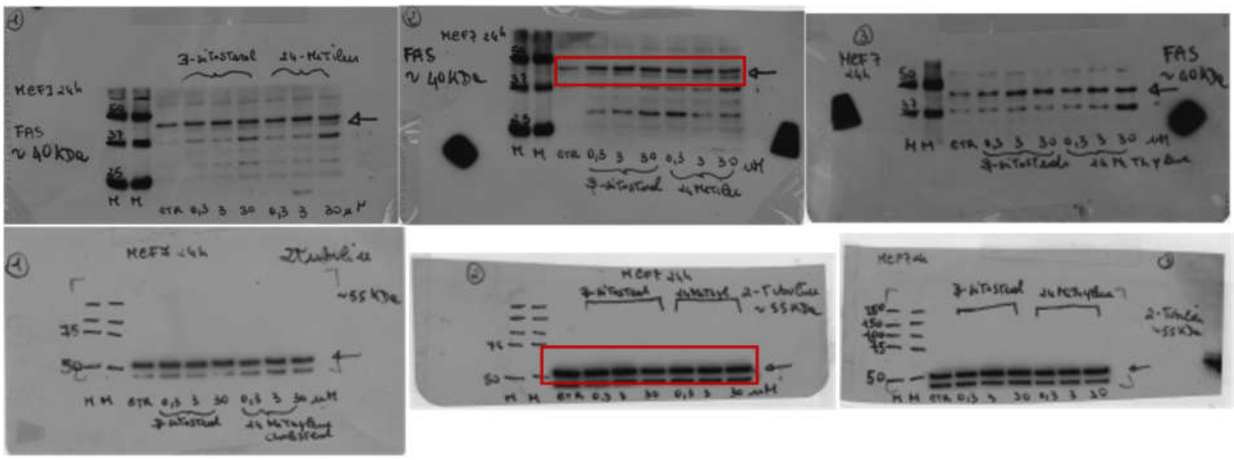

Figure S21. Original blots (biological triplicate) for TRAIL (upper panel) in A549 after treatment with β-SIT and 24-MChol at increasing concentrations (0.3-30 μM). Alpha tubulin (lower panel) has been used as loading control. The arrows indicate the reference band corresponding to the marker with the molecular weights used during migration. The red dial indicates the band inserted into the manuscript

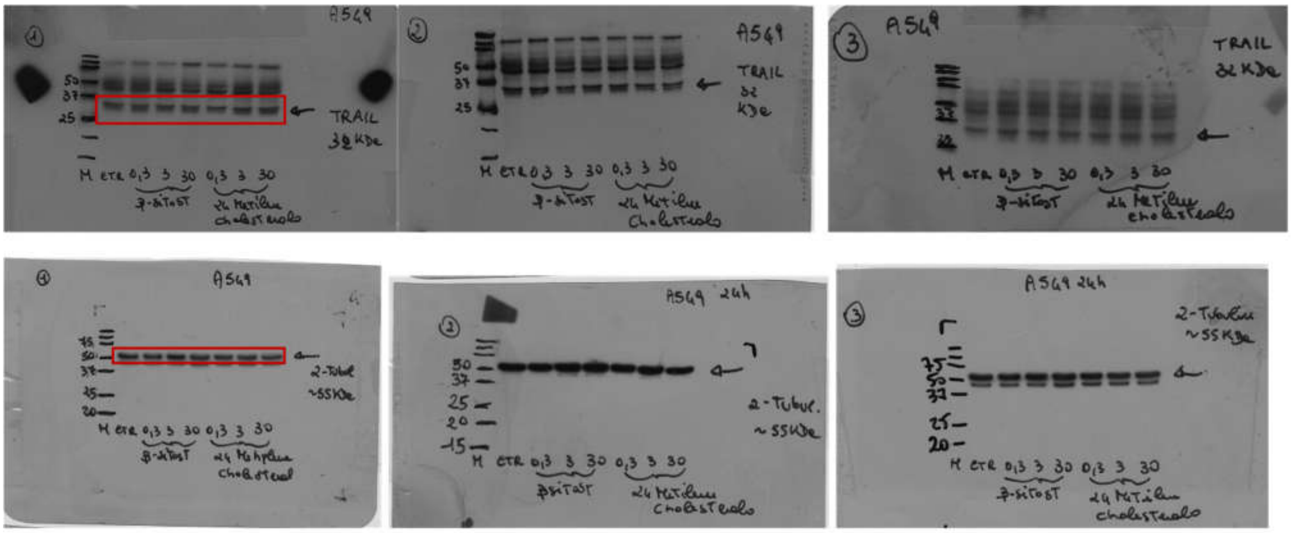

Figure S22. Original blots (biological triplicate) for TRAIL (upper panel) in MCF7 after treatment with β-SIT and 24-MChol at increasing concentrations (0.3-30 μM). Alpha tubulin (lower panel) has been used as loading control. The arrows indicate the reference band corresponding to the marker with the molecular weights used during migration. The red dial indicates the band inserted into the manuscript

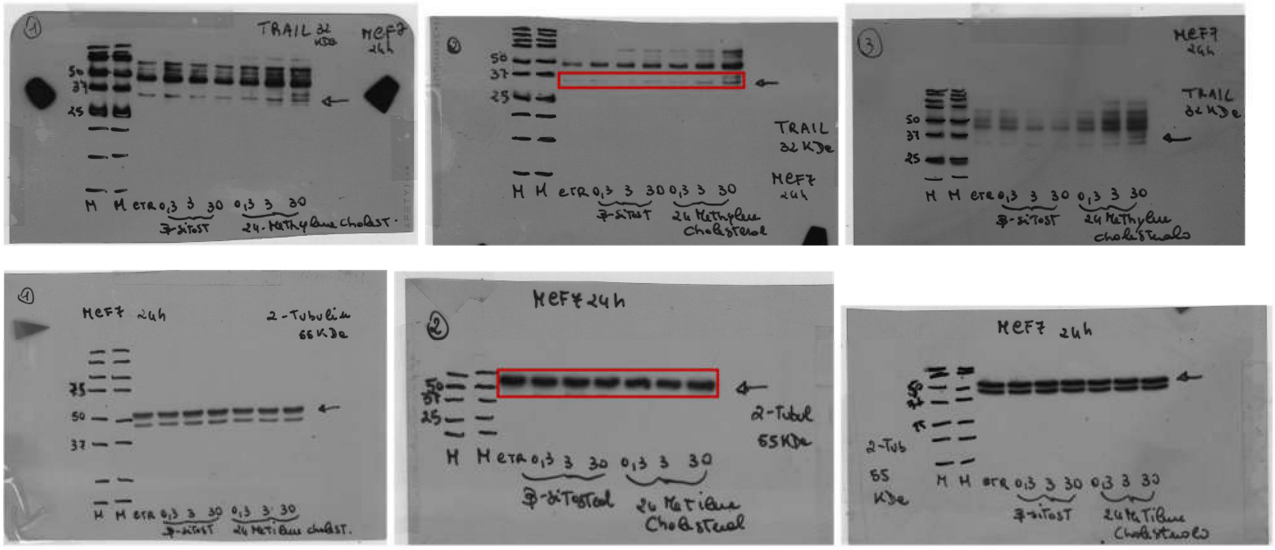

Supplement: Supplementary file 1 [file marinedrugs-20-00595-s001.zip › marinedrugs-1914210-supplementary.pdf]
